# Metal-Free Electrochemical Construction of Oxygen Heterocycles

**DOI:** 10.1021/acs.accounts.6c00306

**Published:** 2026-07-27

**Authors:** Hugo Valdés, Belen Batanero

**Affiliations:** Departamento de Química Orgánica y Química Inorgánica, Instituto de Investigación Química “Andrés M. del Río” (IQAR), Facultad de Ciencias, Edificio de Facultad de Farmacia, 16720 Universidad de Alcalá, Alcalá de Henares, 28805 Madrid, Spain

## Abstract

Oxygen heterocycles are ubiquitous across chemistry—from epoxides that underpin bulk materials and industrial manufacturing to five- and six-membered rings that populate natural products, fragrances, agrochemicals, dyes, and modern medicines. Yet, compared with N-heterocycles, O-heterocycles have historically received less systematic attention in synthetic-method development. One reason is that oxygen’s higher electronegativity often renders these frameworks more reactive and less straightforward to control under conventional conditions. Organic electrosynthesis provides a compelling platform to address this challenge: by using electrons as traceless redox reagents and controlling reactivity through potential or current, electrochemistry enables access to high-energy intermediates (radical-anions, radical-cations, “active halogen”, superoxide) under mild conditions and often without stoichiometric oxidants or reductants. This Account summarizes electrosynthetic conceptions that repeatedly enable the construction—and, in several cases, editing—of oxygen heterocycles spanning three-, five-, and six-membered rings.

We first discuss three-membered oxiranes, emphasizing cathodic manifolds that resemble Darzens-type closures initiated by C–X bond reduction and electrogenerated bases (EGBs), as well as solvent-enabled radical pathways that become accessible only upon reaching the dianion state of 1,2-dicarbonyls. These mechanistic thresholds are conveniently diagnosed by cyclic voltammetry (CV), which serves throughout the Account as a fingerprint for identifying when productive electron-transfer pathways switch on. Complementary anodic epoxidations proceed via mediator-driven oxygen transfer, including iodide-derived “active iodine”.

We then survey five-membered O-heterocycles (furans, dihydrofurans, butenolides, and mixed O,N/O,S rings), highlighting how cathodic and anodic routes deliver distinct synthetic leverage. Representative advances include anodic dimerizations to bioactive butenolides, dearomative spirocyclizations via chalcogen radicals, oxidative [3 + 2] annulations to dihydrofurans, and mediator-enabled cyclizations (TEMPO, triarylamines, iodide) that convert simple feedstocks into densely functionalized heterocycles with high selectivity.

Finally, we address six-membered O-heterocycles-coumarins/δ-lactones, dioxane/dioxine frameworks, and O,N/O,S systems, where electrochemistry enables both ring construction and ring remodeling. Cathodic pathways involving electrogenerated superoxide can trigger Baeyer–Villiger/Dakin-type lactone formation and ring expansion to isocoumarins, while anodic oxidation in terpenoid settings can proceed through carbocationic pathways that induce rearrangement, ring contraction, or lactonization. Beyond ring construction, electrochemistry uniquely enables skeletal editing—most notably redox-triggered ring expansions to isocoumarins and related lactones—by accessing rearrangement-prone radical anions and cationic intermediates under mild conditions. Across ring sizes, a recurring theme is that solvents and electrolytes are often reactive partners: chlorinated media (e.g., dichloromethane, 1,2-dichloroethane) can be electroactivated to generate radical or electrophilic equivalents that unlock otherwise inaccessible cyclizations, an idea that points toward constructive upgrading of chlorinated starting materials into higher-value heterocycles.

Collectively, the examples herein position electrosynthesis as a sustainable design paradigm for oxygen-heterocycle synthesis: it replaces hazardous reagents with electrons, enables mechanistically informed selectivity via CV, and is naturally compatible with flow electrochemistry for improved safety, scalability, and precise control of short-lived intermediates. These features should accelerate future reaction discovery, late-stage ring editing, and practical deployment of O-heterocycles in medicinal and materials chemistry.

## Graphical abstract

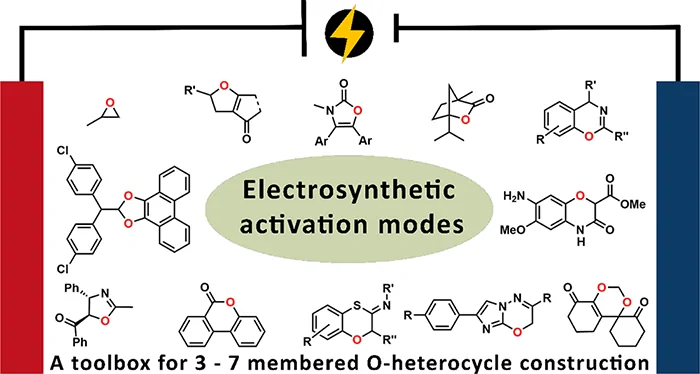

## Key References

-

Batanero, B. ; Salardon, N. ; Prieto-Garcés, E. ; Herrera, L. ; Er-Ryhy, S. ; Quirós, M. T. ; Gómez-Casanova, N. ; Heredero-Bermejo, I. ; Copa-Patiño, J. L. Electrosynthesis of dimeric butenolides by C-C-homocoupling in the oxidation of 2,4-diarylfurans under aqueous conditions. iScience 2024, 27­(9), 110765–110782 10.1016/j.isci.2024.110765 39286499 PMC11404206.^1^ Anodic furan oxidation enables radical homocoupling to bioactive dimeric butenolides under sustainable aqueous conditions.
-

Quintanilla, M. G. ; Pérez, I. ; de Francisco, B. ; Martin, A. ; Salardon, N. ; de la Fuente, J. L. ; Batanero, B. New insights into the phenacyl bromide electroreduction: Unambiguously characterized 5,5́-bis­(3,5-diaryl-2­(5H)-furanone). Anodic oxidation of 2,4-diphenylfuran. J. Electroanal. Chem. 2021, 892, 115265 10.1016/j.jelechem.2021.115265 .^2^ Cathodic phenacyl bromide reduction affords 3,4-epoxy-1,3-diaryl-butan-1-one that evolves to 2,4-diarylfurans under heating. The latter are further diverted into oxidative dimerization to X-ray characterized butenolide frameworks. Plausible mechanism proposals are suggested and discussed.
-

Elinson, M. N. ; Sokolova, O. O. ; Korshunov, A. D. ; Barba, F. ; Batanero, B. Electrocatalytic Cascade Reaction of Aldehydes and 4-Hydroxy-6-methyl-2-pyran-2-one. Electrocatalysis 2018, 9­(5), 602–607 10.1007/s12678-018-0470-6 .^3^ Electrocatalytically triggered cascade annulation provides a mild and efficient entry to coumarin scaffolds.
-

Batanero, B. ; Barba, F. ; Martin, A. One-pot formation of 1,3,4-oxadiazol-2­(3H)-ones and dibenzo­[c,e]­azepines by concomitant cathodic reduction of diazonium salts and phenanthrenequinones. J. Org. Chem. 2013, 78­(18), 9477–9481 10.1021/jo401264w 23957625 .^4^ This electrochemical reduction generates solvent- and electrolyte-controlled radical pathways that deliver O,N-five membered heterocycles in one pot.

## 1 Introduction

Heterocycles are pervasive in secondary metabolites, from toxins to pigments, and in essential biomacromolecules;^5–7^ for example, ribose sugars in nucleotides define the backbone of nucleic acids. Because molecular structure is closely linked to function, heterocyclic compounds remain central to biotechnology, medicinal chemistry, and drug discovery, and both natural and synthetic heterocycles are deeply embedded in modern therapeutics.

Compared with nitrogen heterocycles, oxygen heterocycles—particularly five- and six-membered rings—have received far less sustained attention, despite their broad relevance across chemistry and materials science. Nitrogen heterocycles are more abundant in natural products and approved drugs, driving a richer synthetic and mechanistic literature. By contrast, oxygen heterocycles, whether aromatic or nonaromatic, remain less explored despite offering distinctive physicochemical properties and opportunities in molecular design. This imbalance likely stems partly from oxygen’s higher electronegativity, which often renders O-heterocycles comparatively less aromatic and less stable (e.g., furan or pyran) than their nitrogen analogues (pyrrole and pyridine, respectively) under several reaction conditions. These same features, however, underpin their value in pharmaceuticals,^8–11^ dyes,^12^ agrochemicals,^13,14^ cosmetics, fragrances,^15–17^ and functional materials.^18^ Developing efficient, selective, and sustainable routes to structurally diverse oxygen heterocycles is therefore both timely and enabling.^19^

Electrochemistry is a particularly powerful platform for organic synthesis.^20,21^ By generating highly reactive intermediates at the anode or cathode under controlled conditions, electrochemistry unlocks reactivity that is often inaccessible or inefficient with conventional redox reagents.^22^ Its mechanistic landscape can also be probed by tools such as cyclic voltammetry and, in appropriate cases, electron paramagnetic resonance, enabling close links between redox behavior, intermediate formation, and synthetic outcome. Several recent reviews have compiled and critically framed electrochemical strategies for heterocycle synthesis from complementary perspectives.^23–27^

Rather than assembling an exhaustive catalog of methods,^28–36^ this Account is organized around the recurring electrosynthetic logic that enables oxygen–heterocycle construction. Across ring sizes, successful strategies repeatedly rely on indirect electrolysis (electrocatalysis), paired electrosynthesis, use of electrogenerated bases (EGB), or *in situ* electrosynthesized reagent processes that provide reactive intermediates on demand and guide chemoselective cyclization without stoichiometric oxidants or reductants. To make these patterns readily comparable, we discuss oxygen heterocycles by increasing ring size and organize representative examples into anodic and cathodic pathways, highlighting mechanistic fingerprints that connect redox events to productive ring formation and selectivity.

## 2 Electrosynthesis of Three-Membered O-Heterocycles

Despite their small size, oxiranes (epoxides) occupy a central place in synthesis and chemical manufacturing. Their electrosynthesis is governed by two complementary reaction manifolds: cathodic routes that construct the epoxide through reductive activation and anodic routes that achieve epoxidation through direct or mediator-enabled oxygen transfer. Across both, electrochemistry provides a powerful selectivity handle by generating reactive intermediates on demand under potential control while suppressing competing pathways.

### 2.1 Cathodic Processes

#### 2.1.1 Reductive Epoxide Construction

Cathodic electrosynthesis of oxiranes was accomplished through several complementary strategies. A frequently encountered pathway resembles a Darzens-type process initiated by cathodic C–X bond reduction: the resulting enolate adds intermolecularly to a 2-haloketone, and subsequent intramolecular substitution furnishes ketoepoxides (Scheme 1a).^2,37^

**Scheme 1:**
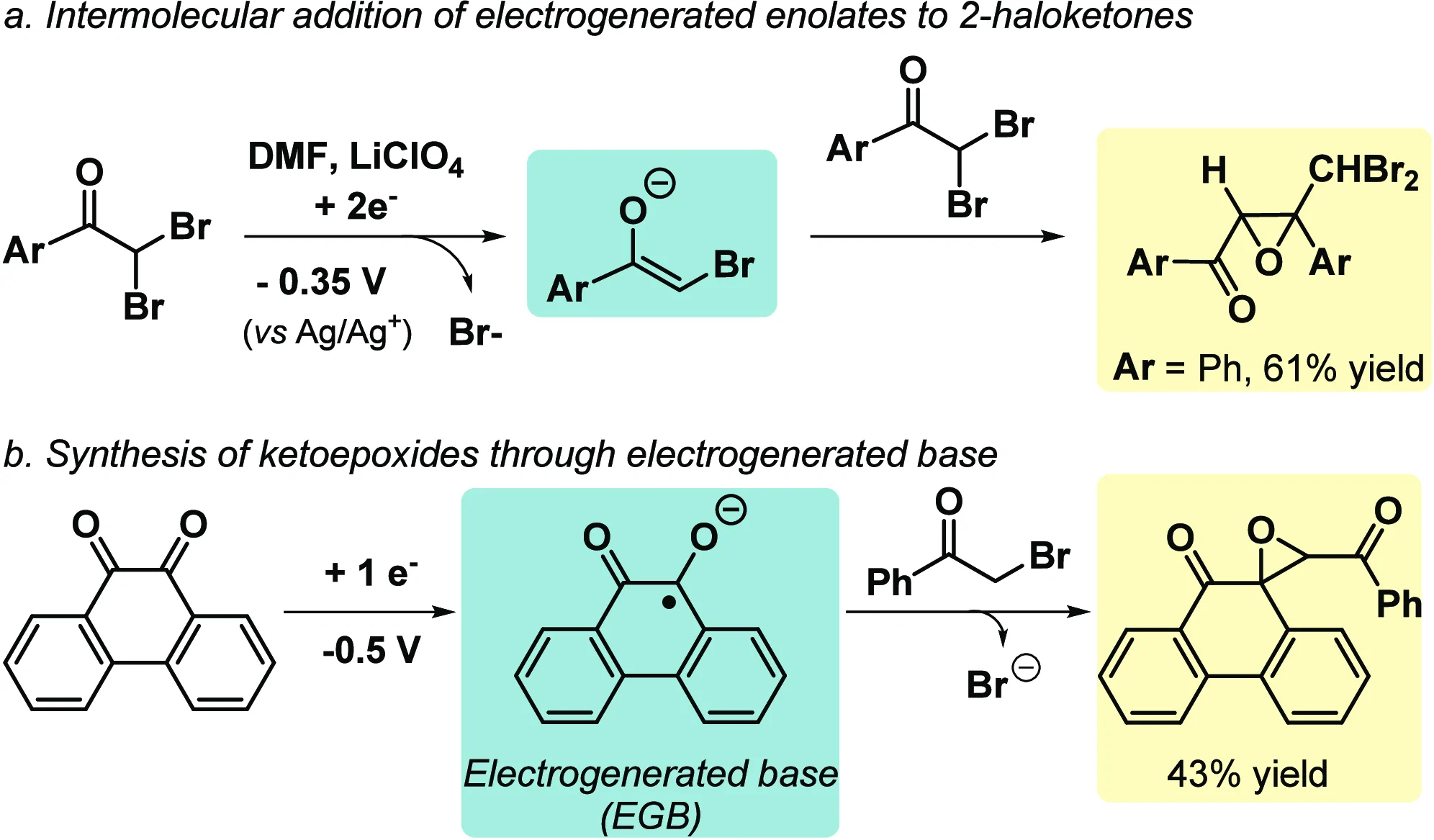
a) Cathodic Darzens-Type Epoxide Formation via Enolate Generation, Intermolecular Addition, and Ring Closure and b) Related Ketoepoxide Synthesis Enabled by Quinone-Derived Electrogenerated Bases

Related ketoepoxides can also be obtained when quinones are reduced to their radical anions, which act as EGBs in reactions with phenacyl halides (Scheme 1b).^38^ In contrast, a distinct pathway operates under concomitant cathodic reduction of arene diazonium salts and quinones in DMF, where C–C radical coupling is followed by intramolecular nucleophilic substitution to form the oxirane ring (Scheme 2).^39^ Under these conditions, reduction of the benzenediazonium salt generates an aryl radical that can abstract a hydrogen atom from 1,2-dichloroethane, producing benzene and a 1,2-dichloroethyl radical. The latter is trapped by the EGB and, through an intramolecular nucleophilic substitution, furnishes oxirane in 14% yield. Notably, when proton sources are present, oxirane formation is suppressed; instead, the corresponding alcohol is formed in 67% yield.

**Scheme 2:**
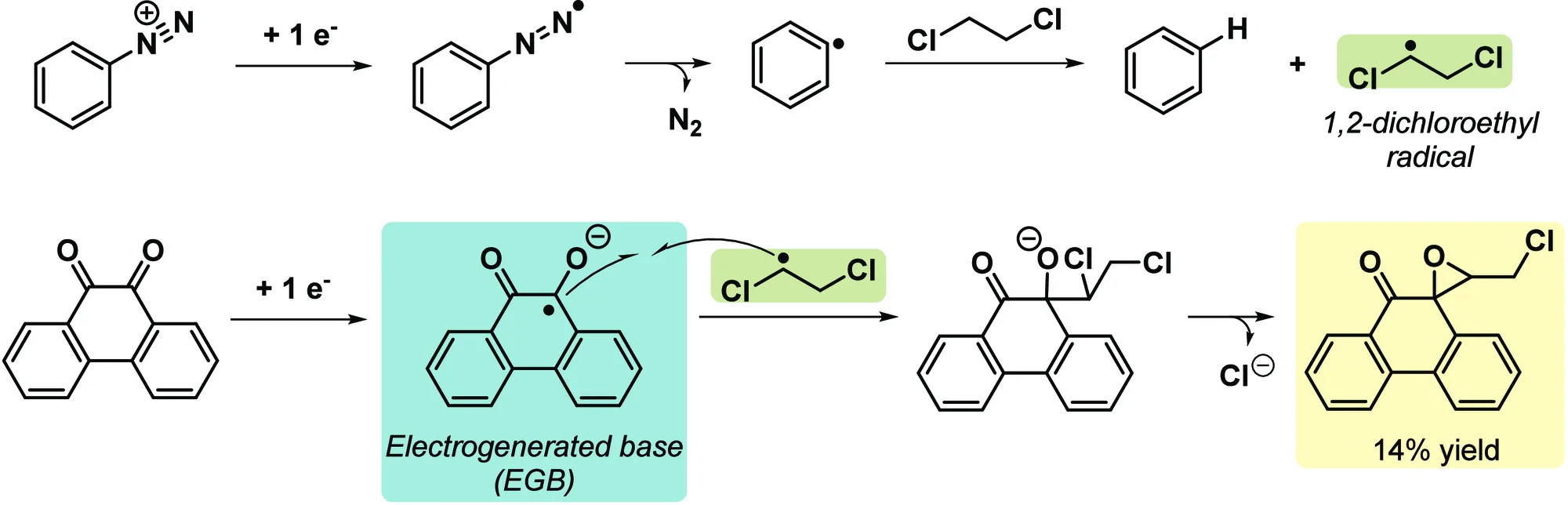
Oxirane Formation *via* an EGB and a 1,2-Dichloroethyl Radical Generated from 1,2-Dichloroethane under Cathodic Conditions

An additional electrochemical approach leverages electrogenerated dianions of 1,2-dicarbonyl substrates to promote homogeneous electron transfer to chlorinated solvents.^40^ The resulting solvent-derived radicals engage in coupling and, after nucleophilic substitution, deliver ketoepoxides (Scheme 3). Crucially, this homogeneous electron-transfer pathway becomes operative only when electrolysis is performed at potentials corresponding to the second reduction event of the 1,2-dicarbonyl substrate (upon formation of the dianion). Figure 1 compares the cyclic voltammograms of 9,10-phenanthrenequinone and benzil (2.0 mM) recorded in TCE/MeCN (3:1) containing 0.1 M Bu_4_NClO_4_ (TBAP) as SSE (vs Ag/Ag^+^; scan rate 100 mV/s; Hg cathode, Pt anode). 9,10-Phenanthrenequinone shows a second cathodic peak potential (Epc2 = – 1.18 V), whereas benzil exhibits only one cathodic peak (Epc = – 1.27 V) under these conditions. Thus, 9,10-phenanthrenequinone reaches the dianion-accessible regime at a less negative potential than benzil. This behavior further illustrates how CV can be used to anticipate the onset of productive solvent-activation chemistry.

**Scheme 3:**
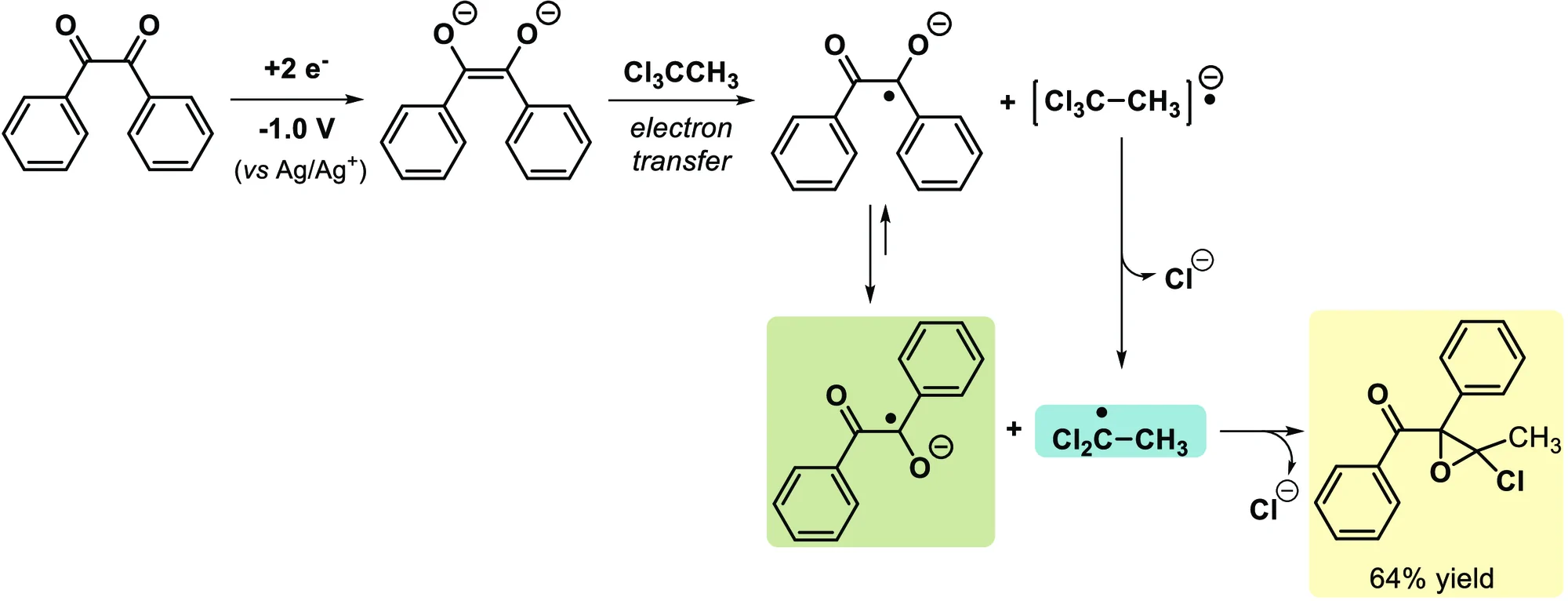
Ketoepoxide Formation via Electrogenerated 1,2-Dicarbonyl Compound Dianions and Solvent-Derived Radicals

**Figure 1:**
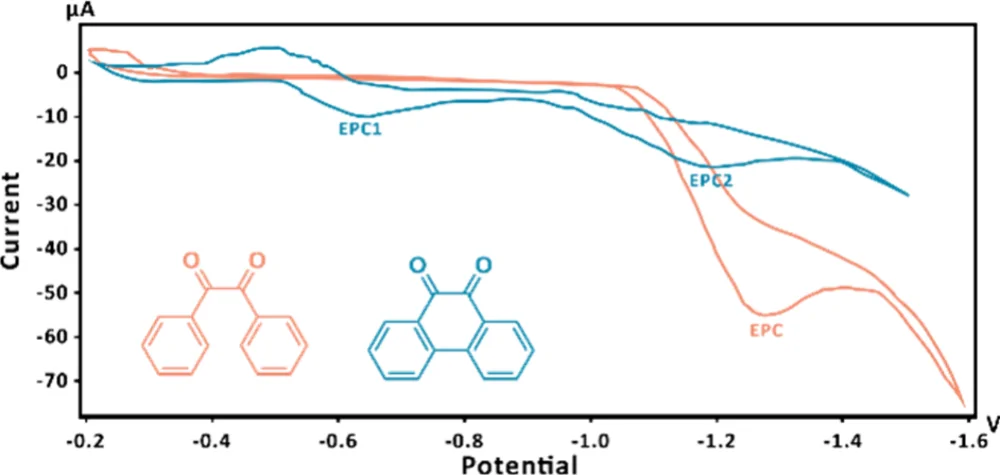
Cyclic voltammetry of 9,10-phenanthrenequinone (blue) and benzil (orange) at 2.0 mM in TCE/MeCN (3:1, v/v) with 0.1 M TBAP as SSE; scan rate = 100 mV/s. E (V, vs Ag/Ag^+^).

Collectively, cathodic epoxide formation is dictated when enolate- or dianion-like nucleophiles become accessible-thresholds that can be anticipated by CV and exploited to achieve chemoselective Darzens-type ring closure.

### 2.2 Anodic Processes: Oxidative Epoxidation

#### 2.2.1 Direct and Mediated Anodic Epoxidation

Oxiranes can be accessed under anodic conditions either by direct substrate oxidation or, more commonly, through mediator-driven oxygen-transfer pathways. An example is the electrooxidation of ketones in MeOH/NaCN in the presence of KI at a Pt anode, which furnishes oxiranes (Scheme 4).^41^ The transformation is proposed to be initiated by electrophilic attack of an electrogenerated “active iodine” species (I^+^) on the enolized carbonyl, thereby priming the system for subsequent ring closure.

**Scheme 4:**
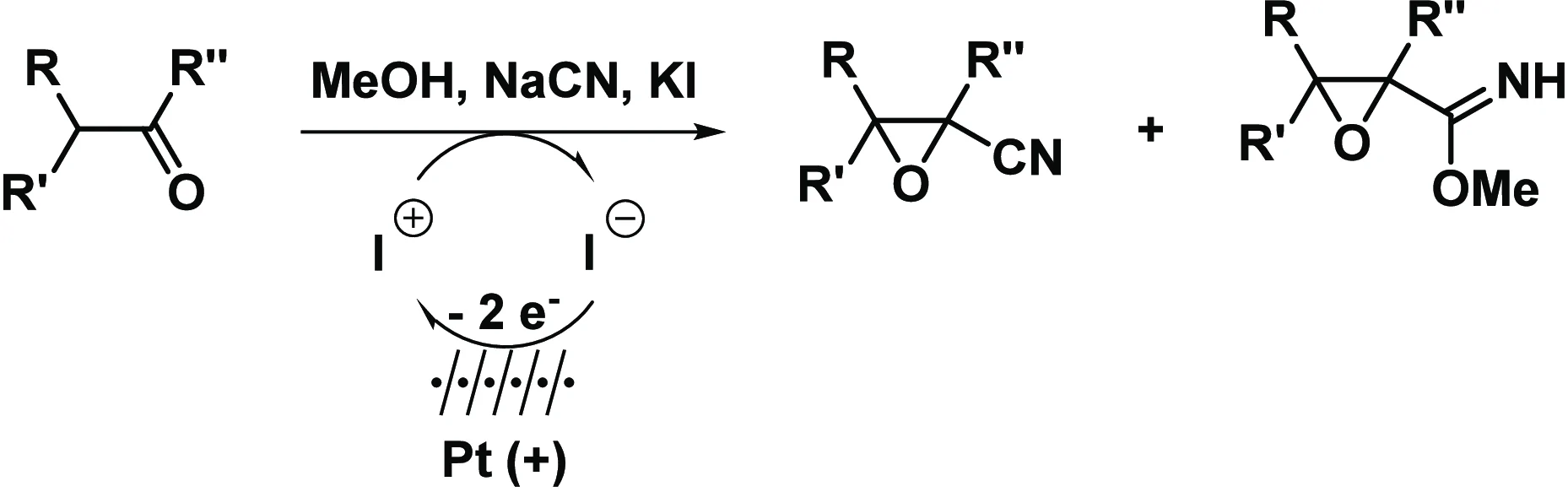
Iodide-Mediated Anodic Epoxidation of Ketones

Halide mediation was also applied to the regioselective electrochemical epoxidation of polyisoprenoids using an MeCN-THF-H_2_O/NaBr system (SSE) at Pt electrodes.^42^ Beyond halogen-based mediators, anodic epoxidation of olefins can be realized through indirect electrosynthesis using high-valent oxidants generated in situ, including Ag­(III)-oxo bis­(2,2′-bipyridine) complexes^43^ and Mn­(III) porphyrins^44^ under electrocatalytic conditions (often in conjunction with electron-transfer mediators such as methylene blue).

At the industrial level, the conversion of propylene to propylene oxide exemplifies chloride-catalyzed indirect electrosynthesis and can be viewed as a convergent paired electrolysis, in which both electrodes generate the reactive species required to form the epoxide (Scheme 5).^45^ At the anode, Cl^–^ is oxidized to Cl_2_, which reacts with water to generate HClO; this adds to propylene to give 1-chloro-2-propanol, which is subsequently cyclized to propylene oxide by OH^–^ generated at the cathode via water reduction. This paired process highlights a central advantage of electrosynthesis: both electrodes can generate complementary reactive partners in situ, increasing efficiency and sustainability.

**Scheme 5:**
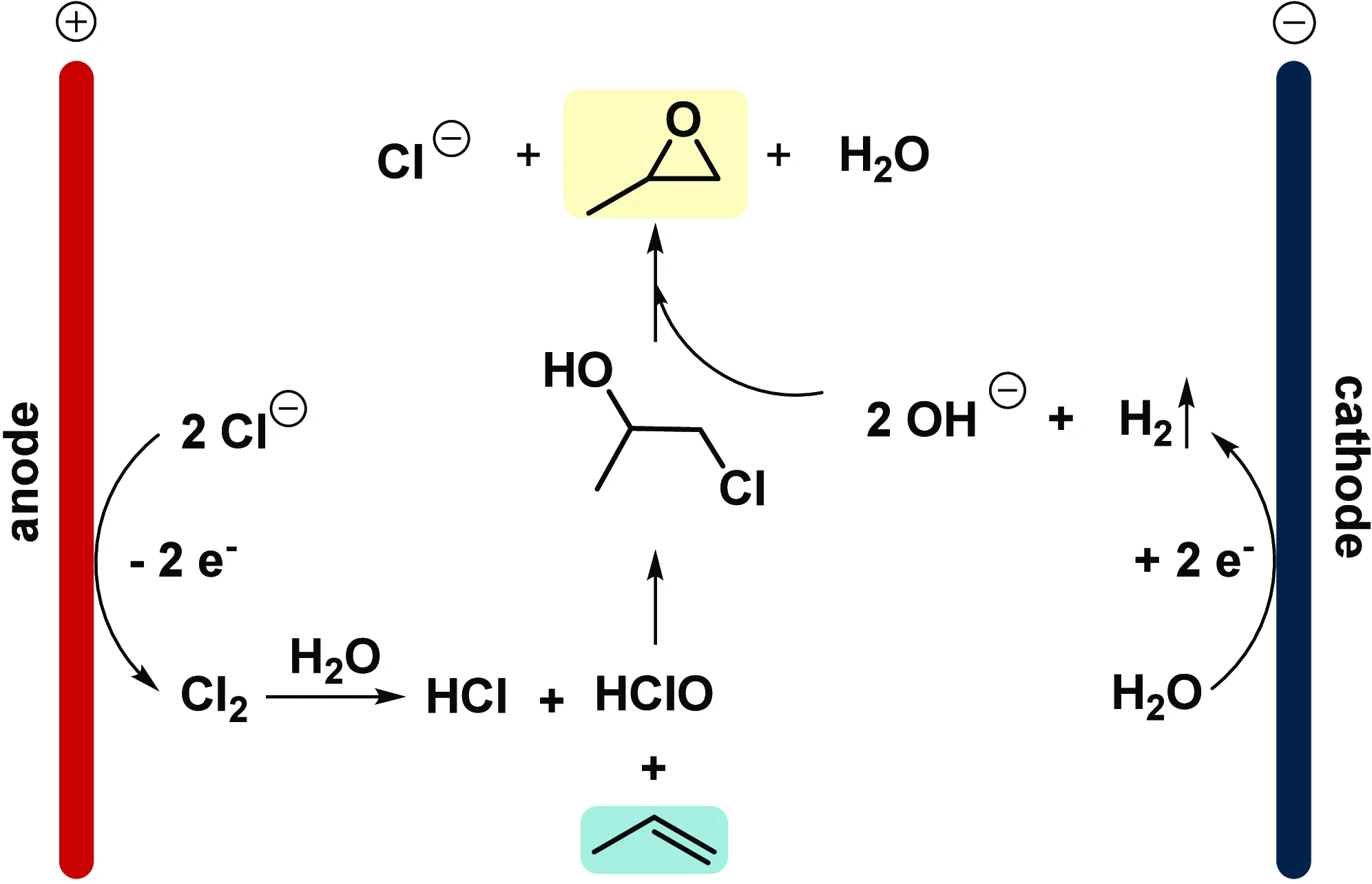
Convergent Paired Electrolysis for Propylene Oxide Synthesis Mediated by Chloride

Overall, anodic epoxidation showcases how redox mediators translate electrode potentials into practical oxygen-transfer reactivity, enabling selective epoxide formation and, in paired settings, industrially relevant processes with minimal reagent burden.

## 3 Electrosynthesis of Five-Membered O-Heterocycles

Five-membered O-heterocycles are exceptionally versatile motifs at the interface of synthesis, manufacturing, and the life sciences. Electrochemistry provides a particularly effective platform for their construction by generating radical, anionic, and cationic intermediates on demand under mild conditions. Across this section, recurring strategies include electrogenerated bases, mediator-enabled anodic radical chemistry, and active solvent/supporting-electrolyte participation, with cathodic and anodic manifolds offering complementary routes to these valuable frameworks.

### 3.1 Cathodic Pathways to Five-Membered O-Heterocycles

#### 3.1.1 Furans and Furan Derivatives

2,4-Diarylfurans were prepared in good yields by Barba and co-workers via cathodic reduction of phenacyl bromides in DMF.^2,46^ The transformation proceeds through an isolable spiro keto–epoxide intermediate, which upon heating rearranges to the furan. Switching the solvent from DMF to acetone diverts the outcome: reduction of phenacyl bromides furnishes 4-aryl-2-methylfurans.^47^ Along similar lines, 5-amino-4-benzoyl-3-phenylfurans were obtained upon C–Br bond reduction of 2-bromo-2-cyanoacetophenone in a DMF/LiClO_4_ SSE system (Scheme 6).^48^ Collectively, these reactions are consistent with a Darzens-type manifold in which brominated enolates derived from aromatic ketones add to a carbonyl group of the substrate, followed by ring closure and aromatization to the furan scaffold.

**Scheme 6:**
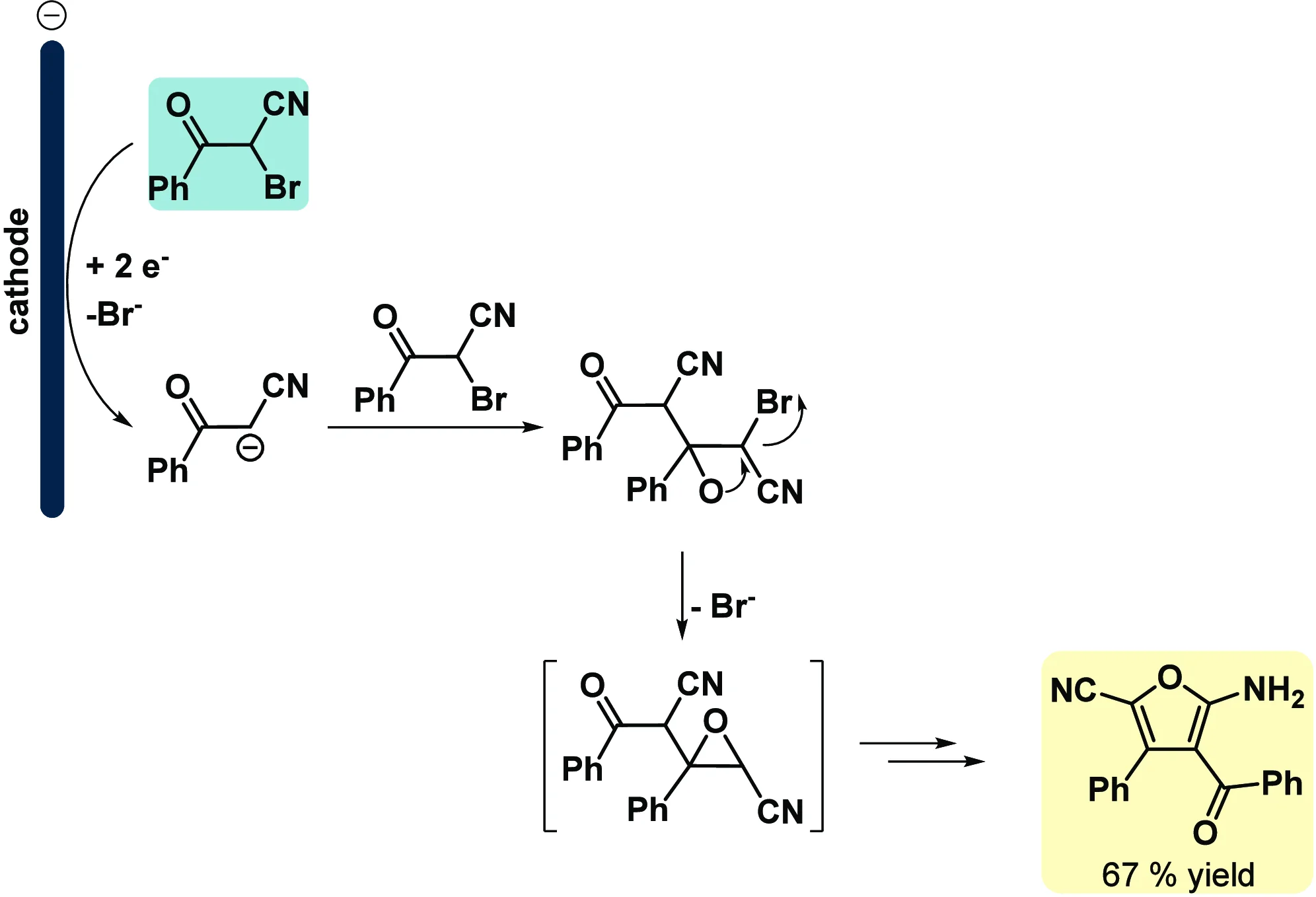
Cathodic Darzens-Type Access to Polysubstituted Furans from Phenacyl Bromides

More structurally complex annulated furans can also be accessed electroreductively. For example, 2,3-bis­(spiro-2-indanyl-1,3-dione)-indeno­[1,2-*b*]­furan-4-one was obtained from the electroreduction of a 2,2-dibrominated 1,3-indandione in dichloromethane (Scheme 7).^49^ In the presence of olefins, cathodic reduction of 2,2-dibromo-1,3-diketones affords [3 + 2] cycloadducts, delivering 2,3-dihydrofuran derivatives with high regioselectivity (Scheme 7).^50^

**Scheme 7:**
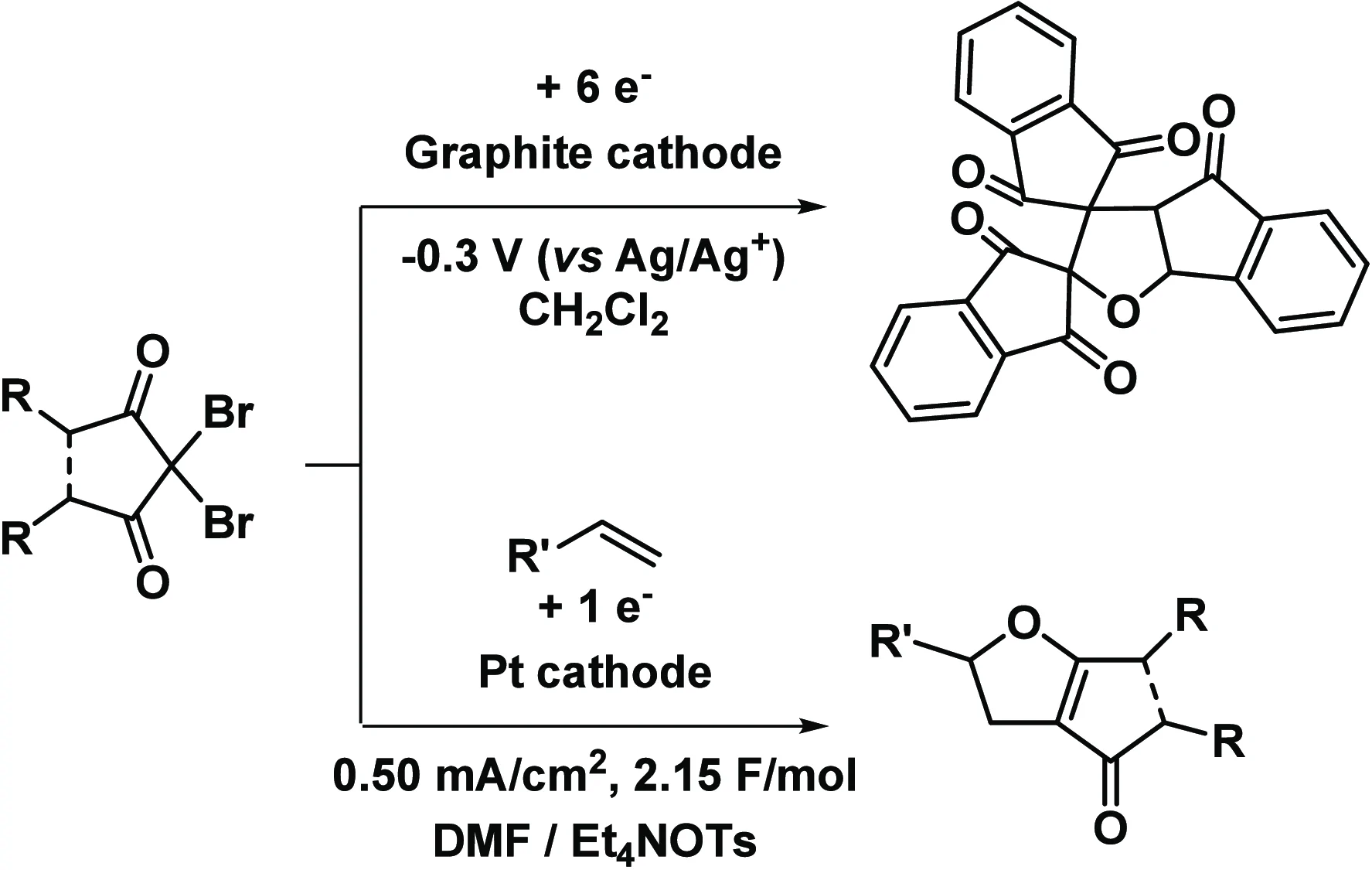
Electroreductive Annulation and Cycloaddition of Gem-Dibrominated 1,3-Dicarbonyls to Access Fused Furans and Dihydrofurans

Radical pathways provide complementary access to saturated rings. Upon reduction of mixtures of alkyl halides and vitamin B_12_ at relatively positive potentials, radical-type intermediates can be generated and trapped by polyunsaturated substrates to furnish tetrahydrofurans (Scheme 8).^51^ In this context, the cobalamin/cobaloxime platform functions as an electrocatalyst that mediates C–Br bond reduction.

**Scheme 8:**
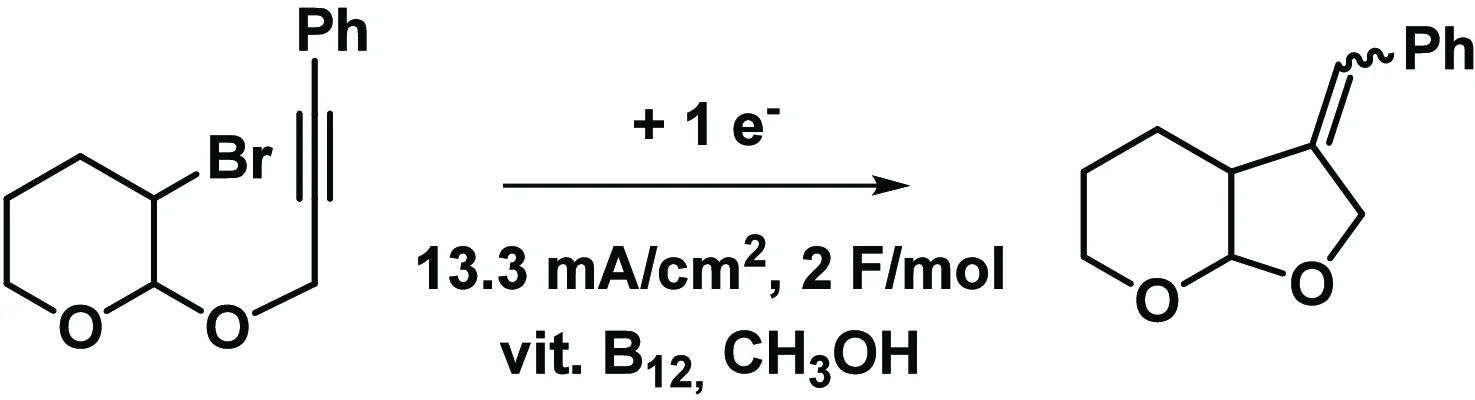
Electrocatalytic C–Br Bond Reduction by Vitamin B_12_ Enabling Radical Cyclization to Tetrahydrofurans

Finally, controlled-potential electrolysis of benzil in acetone/LiClO_4_ at – 1.2 V (vs Ag/Ag^+^) affords 5-methyl-2,3-diphenylfuran.^52^ This outcome was attributed to nucleophilic addition of the acetone enolate to the carbonyl group of benzoin, followed by cyclization/dehydration to the aromatic furan.

#### 3.1.2 1,3-Dioxoles

1,3-Dioxoles can be formed upon cathodic generation of 1,2-dicarbonyl dianions (e.g., from o-quinones) in the presence of chlorinated solvents such as dichloromethane.^53^ This transformation (Scheme 9) is typically interpreted as an indirect solvent activation: electron transfer ultimately produces a chloromethyl radical, which undergoes coupling and subsequent cyclization to deliver the five-membered dioxole ring.

**Scheme 9:**
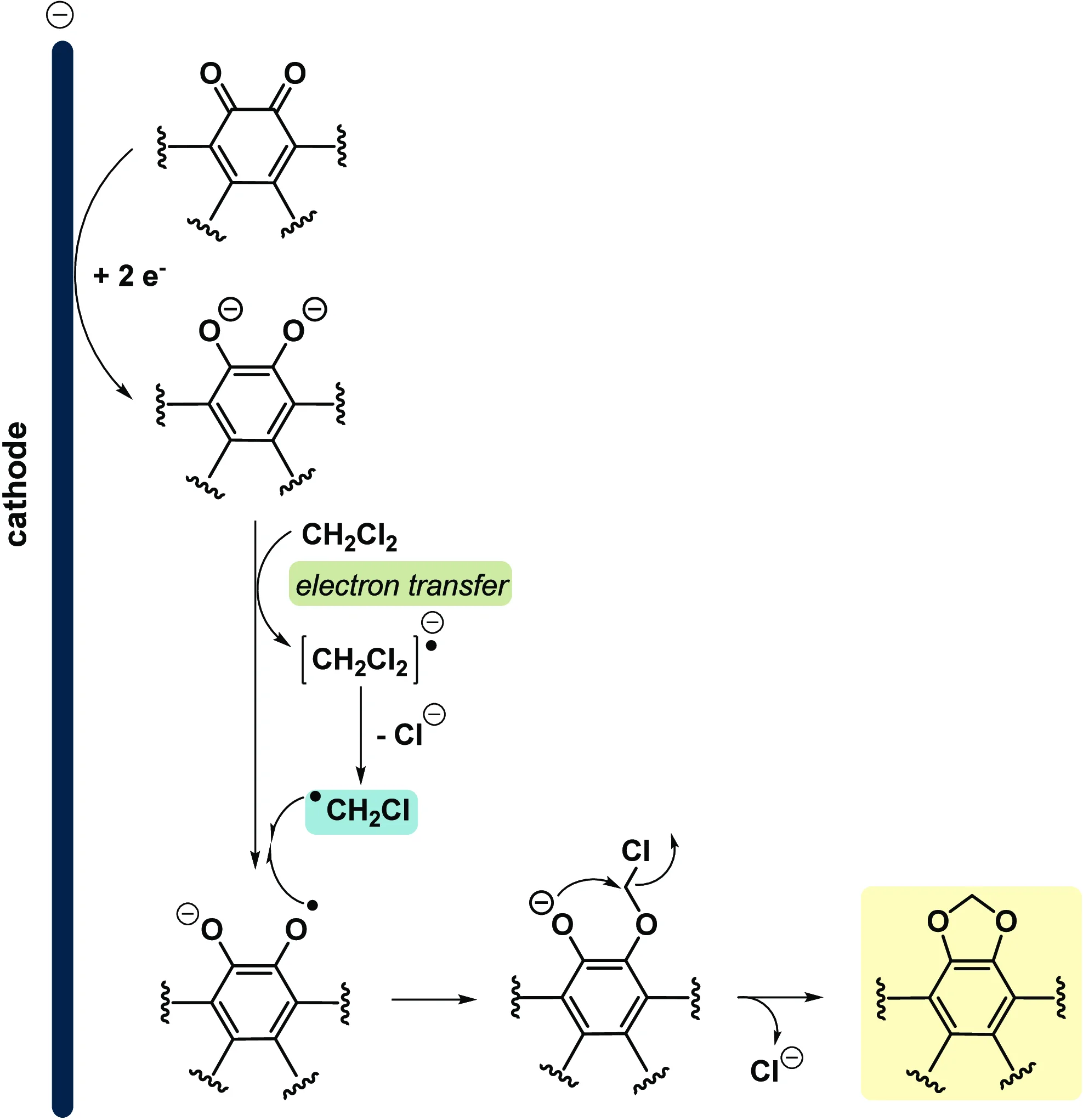
Electroreductive Formation of 1,3-Dioxoles via Quinone Dianions and Chlorinated-Solvent Activation

When 1,1,1-trichloroethane (TCE) is used as the solvent, 2-methyl-1,3-dioxoles are obtained in up to 72% yield from 9,10-phenanthrenequinone. Applying this reactivity to the gem-trichlorinated pesticide DDT enables its conversion into the corresponding 2-alkylated 1,3-dioxole (Scheme 10).^40^ Owing to its structural resemblance to helicene-like frameworks, such products were discussed as potential precursors for OLED-related materials. More recently, cathodic [3 + 2] ring-expansion strategies have extended this logic to the eco-friendly synthesis of 1,3-dioxolanes, cyclic carbonates, and oxazolines.^54^

**Scheme 10:**
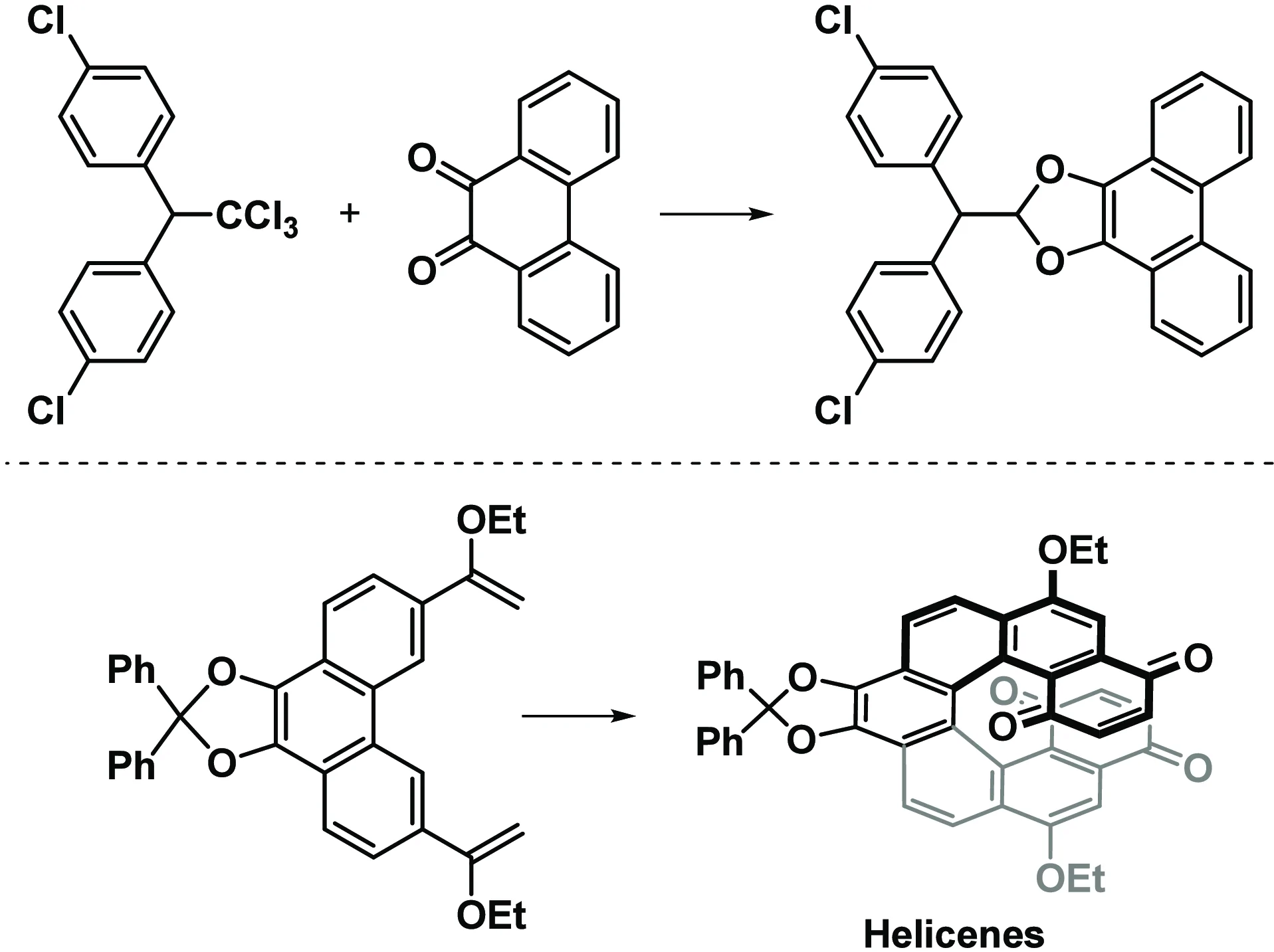
Electrochemical Derivatization of DDT to a Helicene-like 2-Alkylated 1,3-Dioxole Scaffold

A key take-home message is that chlorinated media can be leveraged as constructive partners, serving as radical/electrophile sources under cathodic control, rather than being treated as inert solvents.

#### 3.1.3 Cyclic Carbonates

Electrolysis of 1,2-quinones such as 9,10-phenanthrenequinone in dichloromethane at – 0.5 V (vs Ag/Ag^+^) in the presence of triphosgene as an electrophile furnishes, after consumption of 2 F/mol, phenanthro­[9,10-*d*]­[1,3]­dioxol-2-one in 90% yield (Scheme 11).^29^ The reaction proceeds through successive nucleophilic attacks on the carbonyl groups of triphosgene, accompanied by chloride elimination.

**Scheme 11:**
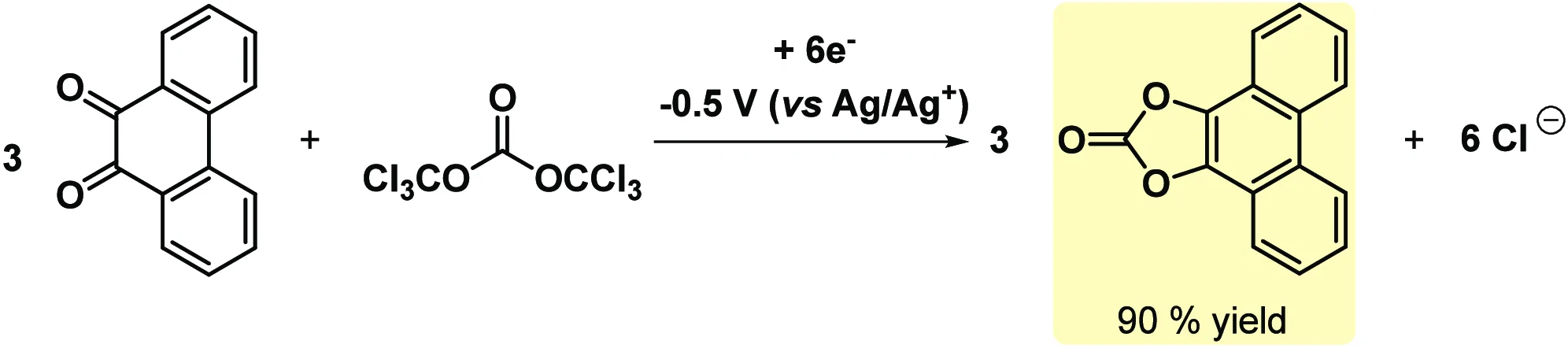
Electrochemical Synthesis of Phenanthro­[9,10-*d*]­[1,3]­dioxol-2-one from 9,10-Phenanthrenequinone and Triphosgene

A complementary electrosynthetic platform enables [3 + 2] ring expansion of epoxides to access 1,3-dioxolanes, oxazolines, and cyclic carbonates via insertion of acetone, acetonitrile, or CO_2_, respectively.^54^ The efficiency of these transformations is markedly enhanced by Lewis-acidic ionic-liquid SSE and by tailored cathodic metal electrocatalysts (e.g., metal-modified carbon-fiber electrodes), which collectively promote substrate activation and productive heteroatom incorporation.

#### 3.1.4 Five-Membered O-Heterocycles with N or S Heteroatoms

4,4-Diphenyl­[1,3]­oxathiolan-5-one was obtained upon cathodic reduction of 2-bromo-2,2-diphenylacetyl bromide in CH_2_Cl_2_/Et_4_NBr at a graphite cathode.^55^ The transformation was proposed to involve in situ generation of hydrogen sulfide from sodium thiosulfate decomposition, which intercepts electrogenerated diphenylketene to furnish the thiocarbonyl-derived heterocycle.

In a distinct cathodic pathway, 1,3,4-oxadiazol-2­(3*H*)-ones can be accessed through concomitant reduction of arenediazonium salts and 9,10-phenanthrenequinone, proceeding via a rearrangement pathway.^4^ Likewise, biologically relevant 1,3-oxazol-2-ones are obtained in good yields when selected 1,2-dicarbonyl compounds are electrolyzed in the presence of arenediazonium salts under controlled-potential conditions in DMF/LiClO_4_ as SSE (Scheme 12).^56^ Benzisoxazoles can also be prepared by cathodic reduction of readily available nitroarenes in an undivided cell under constant-current conditions, providing a direct electroreductive entry to N,O-containing five-membered heterocycles.^57^

**Scheme 12:**
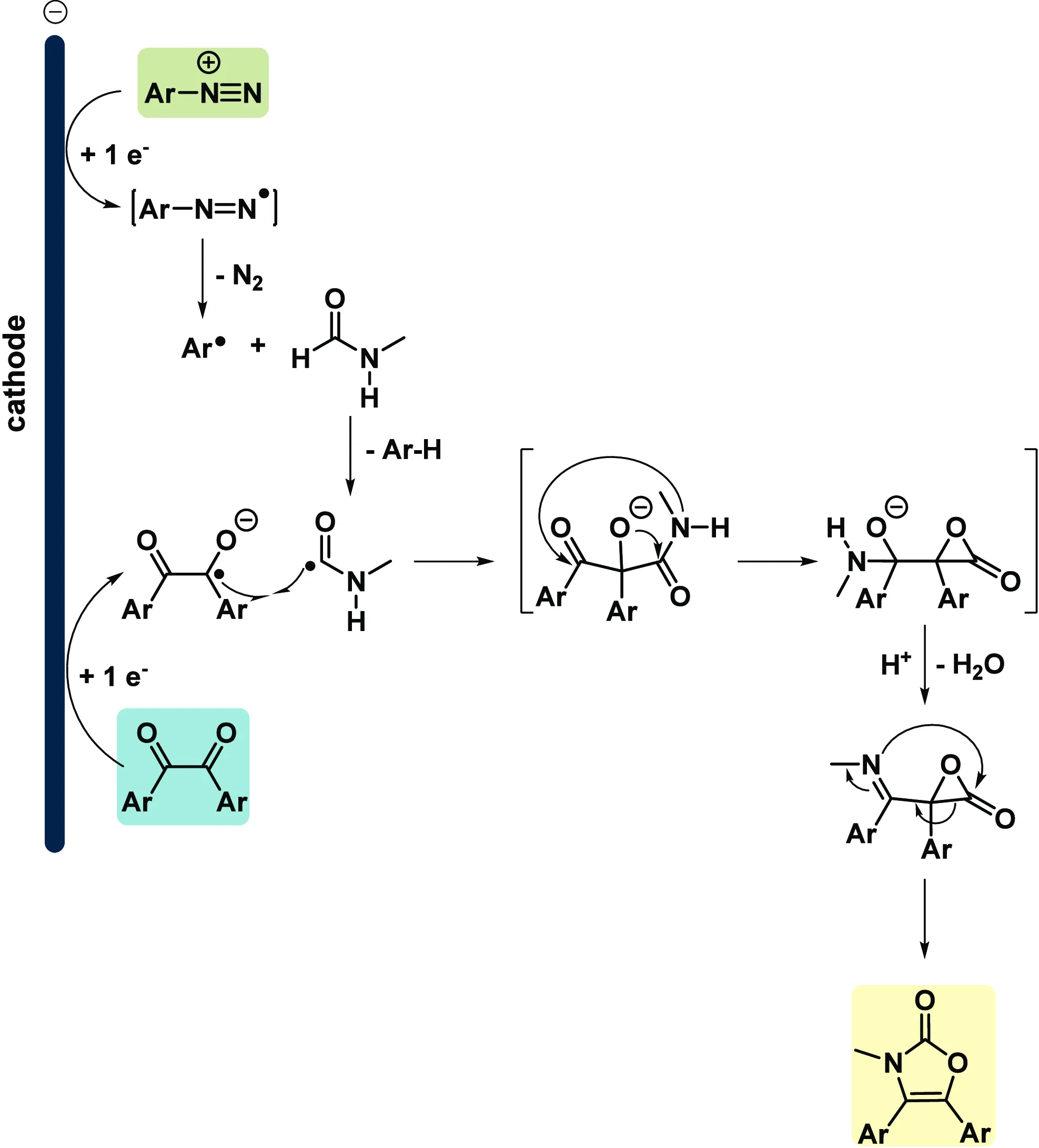
Concomitant Reduction of Arenediazonium Salts and 1,2-Dicarbonyls to Access 1,3-Oxazol-2-ones

Taken together, cathodic routes to five-membered O-heterocycles highlight how reductive triggering, via C–X bond cleavage, EGB formation, or solvent-derived radicals, provides modular access to furans and related rings with reactivity regimes that are often predictable from electroanalytical signatures.

### 3.2 Anodic Pathways to Five-Membered O-Heterocycles

#### 3.2.1 Furans and Derivatives

Electro-oxidative [3 + 2] annulation between 1,3-dicarbonyl compounds and alkenes has been reported to afford acylated dihydrofurans in good yields under basic conditions, using MeCN/HFIP at a carbon rod anode (Scheme 13).^58^ This transformation can be viewed as the anodic counterpart of Yoshida’s electrosynthesis of dihydrofurans. Likewise, 2-aryl-1,5-diols undergo efficient cyclization to 2-aryltetrahydrofurans at a graphite anode in CH_2_Cl_2_/TFE with ^*n*^Bu_4_NClO_4_ at room temperature, likely via mesolytic cleavage followed by intramolecular trapping of a carbocation intermediate.^59^ Isochromene and dihydrobenzofuran derivatives can also be obtained regioselectively from 2-ethynylbenzaldehydes in good yields using silver as a sacrificial electrode, expanding anodic access to benzo-fused O-heterocyclic frameworks.^60^

**Scheme 13:**
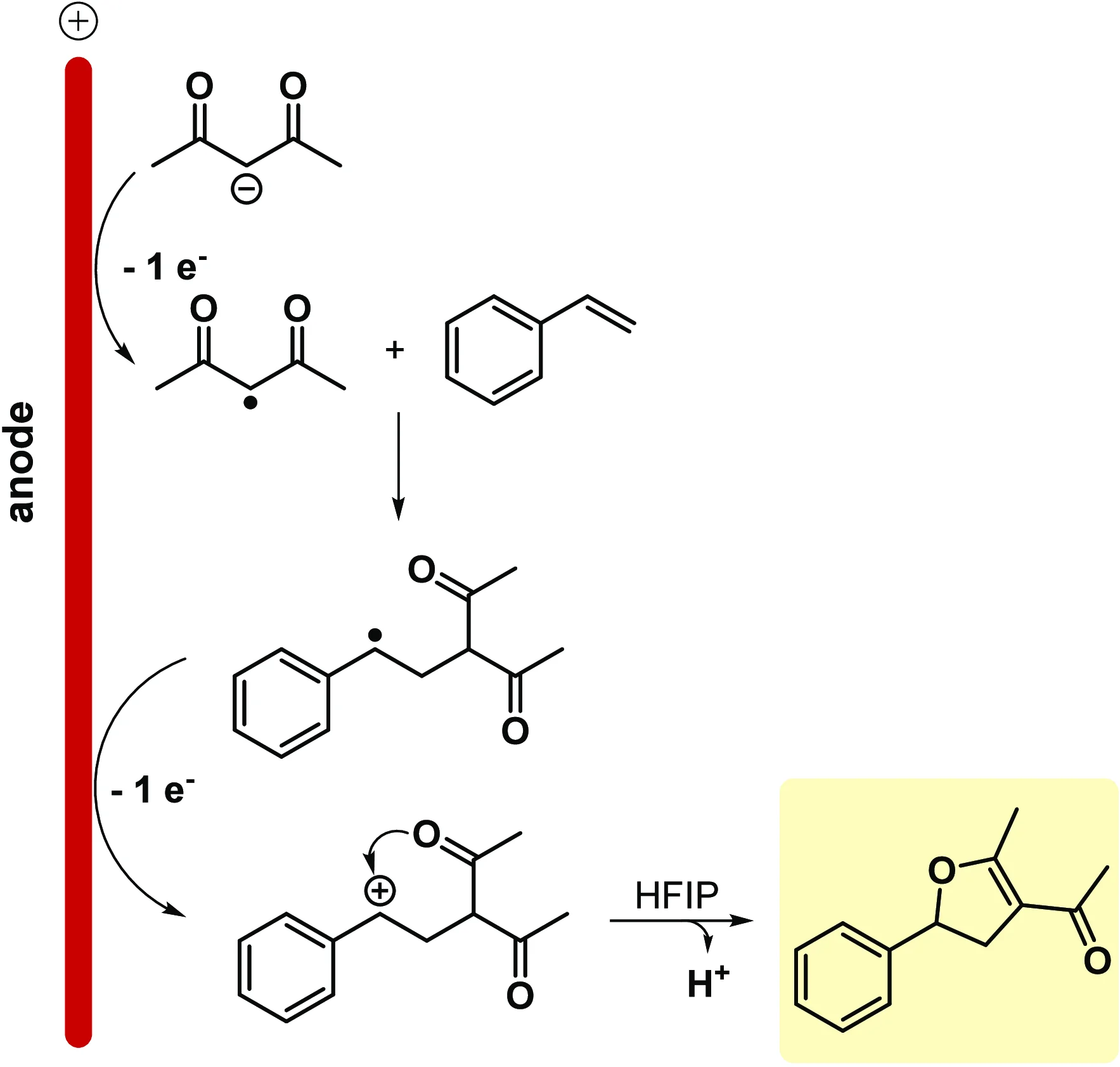
Electro-oxidative [3 + 2] Annulation of 1,3-Dicarbonyl Compounds with Alkenes to Acylated Dihydrofurans

#### 3.2.2 Butyrolactones

A fast and sustainable electrochemical route to diarylated dimeric furan-2­(*5H*)-ones was reported via anodic oxidation of disubstituted furans at room temperature under constant-current conditions in aqueous acetonitrile (Scheme 14).^1^ The resulting dimeric lactones were shown to display biocidal and bactericidal activity.

**Scheme 14:**
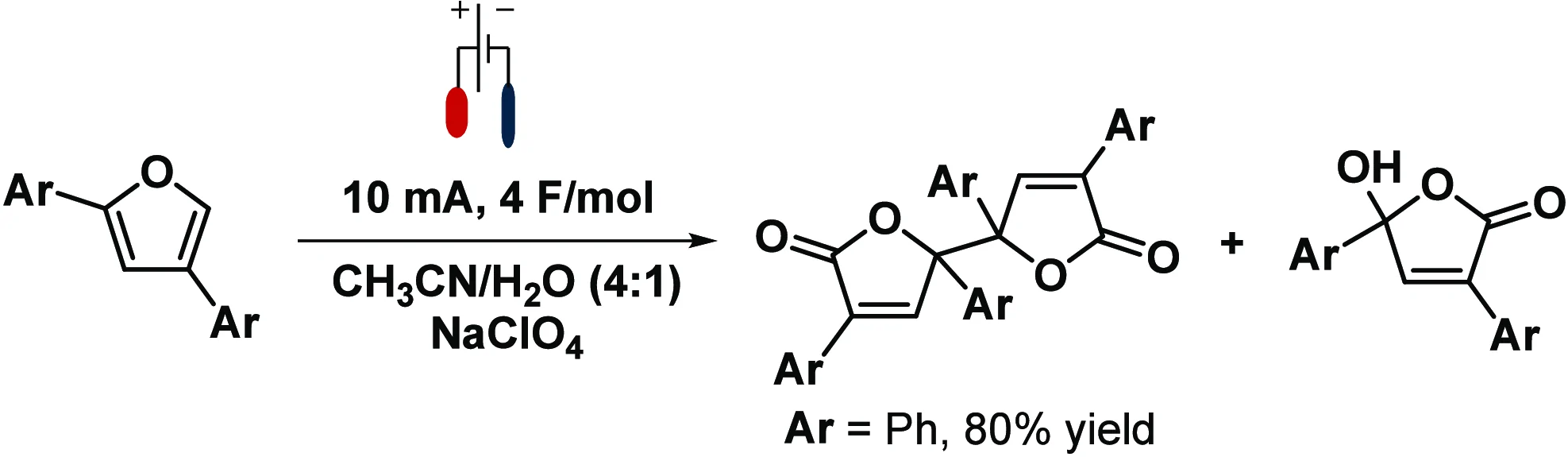
Anodic Dimerization of Diarylated Furans to Dimeric Furan-2­(*5H*)-ones

A practical and green dearomative spirocyclization of alkynes with diselenides was also developed under metal-free and oxidant-free conditions, delivering spiro-butenolides in moderate-to-good yields at a graphite rod anode in MeCN/HFIP using ^*n*^Bu_4_NPF_6_ as SSE.^61^ A radical pathway involving phenylselenyl radicals was proposed. Along similar lines, the anodic oxidation of 2-vinylbenzamides with diselenides to afford selenium-substituted iminoisobenzofurans was described at room temperature in acetonitrile/LiClO_4_, using a microreactor setup.^62^

Regarding aliphatic γ-lactones, a range of butyrolactones can be accessed by anodic oxidation of glutaric acid carboxylates in methanol under constant-current electrolysis (1A), reaching completion within 30 min.^63^ The transformation proceeds through initial heterogeneous electron transfer followed by decarboxylation (Scheme 15). Yields increase with higher α-substitution on the (non-rigid) dicarboxylate precursor.

**Scheme 15:**
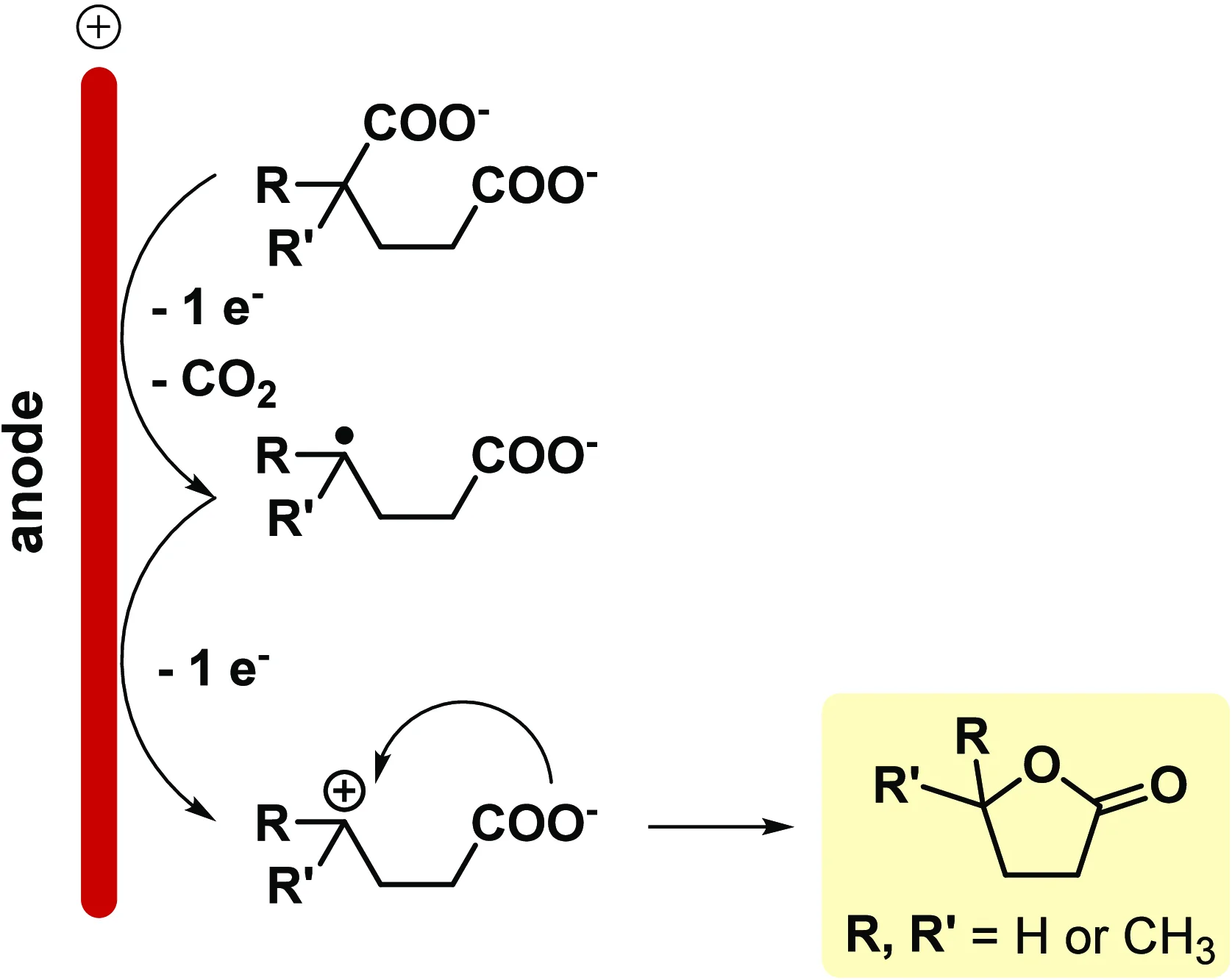
Anodic Decarboxylative Oxidation of Glutarates to γ-Lactones (Butyrolactones)

Related anodic oxidative cyclizations of enol ethers bearing amide trapping groups further show how the reaction medium can redirect cationic intermediates toward furanone formation, with water playing a key role in enabling productive cyclization.^64^

In 2017, an electrochemical C–H lactonization of δ-ketoacids was reported, where ^n^Bu_4_NI served as an iodide mediator/catalyst to furnish furanones in good yields.^65^ Butyrolactones were also prepared via carboxydifluoromethylation of alkenes in MeCN/H_2_O at a Pt anode.^66^ Ferrocene-mediated intermolecular oxidative annulation between malonates and styrenes further illustrates how redox mediators can channel anodic radical chemistry toward functionalized butyrolactone scaffolds.^67^

#### 3.2.3 O,N-Heterocycles and O,S-Heterocycles

Electrosynthesis of 2-oxazolines have received special attention due to their application in synthesis of complex molecules as carboxylic acid protecting group or aromatic ring activation versus nucleophiles.^68^ Trisubstituted 2-oxazolines can be accessed electrochemically via intramolecular C­(sp^3^)–H functionalization using masked oxygen nucleophiles, with KI serving as both catalyst and electrolyte (Scheme 16).^69^

**Scheme 16:**
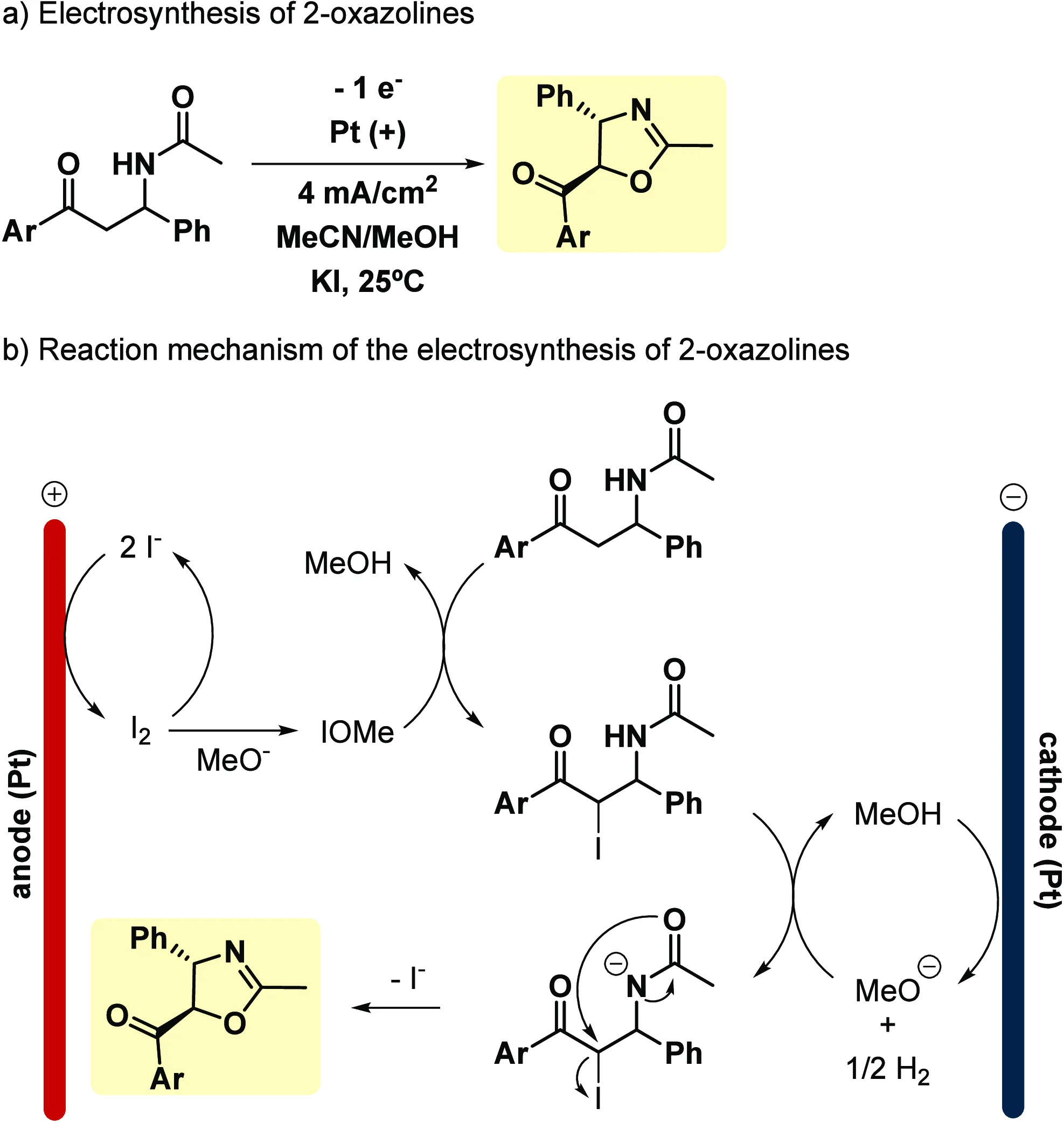
a) KI-Catalyzed Anodic Intramolecular C­(sp^3^)–H Functionalization En Route to Trisubstituted 2-Oxazolines and b) Reaction Mechanism of the Electrosynthesis of 2-Oxazolines

An efficient route to substituted oxazoles proceeds through a triarylamine-mediated electrochemical Ritter-type reaction of ketones with acetonitrile followed by oxidative cyclization, delivering products in good-to-excellent yields with broad functional-group tolerance (Scheme 17).^70^ Direct anodic oxidation of readily available aldoximes provides a complementary entry to isoxazoles in good yields, using an undivided cell and broadly available electrode materials.^71^ An electrochemically promoted oxidative cyclization of 2-alkyn-1-one O-methyloximes with CF_3_SO_2_Na has also been developed, providing direct access to 4-trifluoromethylated isoxazoles.^72^

**Scheme 17:**
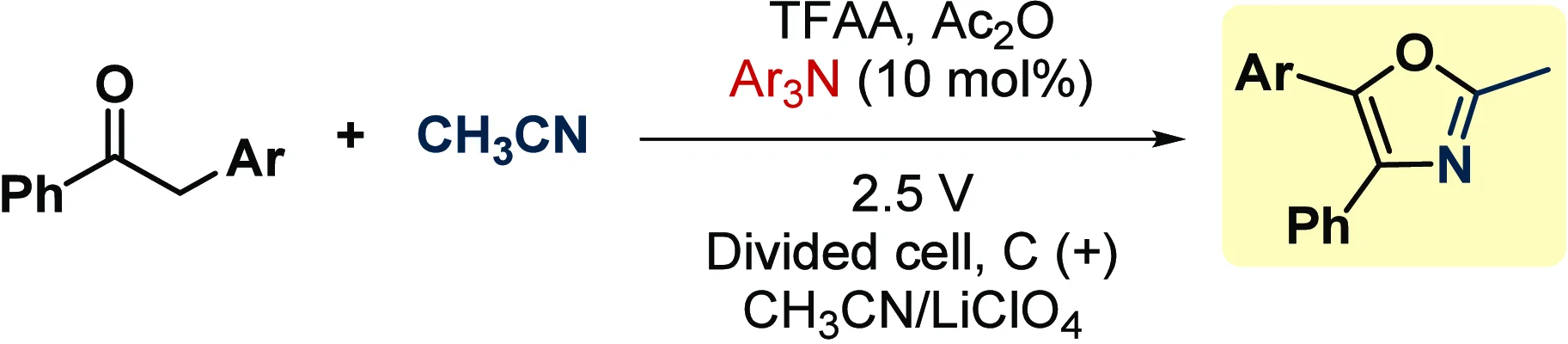
Triarylamine-Mediated Electrochemical Ritter-Type Reaction/Oxidative Cyclization of Ketones with MeCN to Substituted Oxazoles

Oxazol-2-ones were also obtained anodically through TEMPO-mediated cyclization of propargylic carbamates and ureas.^73^ In this reaction, TEMPO operates as a redox mediator to generate amidyl radicals and as an oxygen-atom donor. Likewise, electrogenerated amidyl radicals can undergo intramolecular addition to alkynes, ultimately providing isoxazolidine-fused isoquinolin-1­(2*H*)-ones after a second cyclization event.^74^ Selenium-containing oxazolidinones can also be accessed through electrocatalytic selenocyclization of N-Boc allylamines with diselenides in an undivided cell, proceeding through a hybrid radical/cationic pathway under oxidant-free conditions.^75^

Benzoxazole compounds were prepared via a Shono-type oxidation of N-(2-hydroxyphenyl)­ethyl glycinate at a graphite anode in acetonitrile/^*n*^Bu_4_NBF_4_ in the presence of AcOH (undivided cell).^76^ Highly substituted benzoxazolone derivatives were further accessed through a dehydrogenative cascade that constructs both the benzene and heterocyclic rings, using an RVC anode in AcOH/Et_4_NPF_6_ (SSE) with TFE under heating conditions.^77^ Finally, pharmaceutically relevant symmetric 2,5-disubstituted 1,3,4-oxadiazoles were synthesized in good yields by anodic oxidation of aldehyde N-arylhydrazones in anhydrous MeCN/LiClO_4_ under constant-potential electrolysis in an undivided cell with Pt electrodes.^78^

In sum, anodic manifolds enable five-membered ring construction through mediator-controlled radical and cationic pathways, offering mild and scalable entries to lactones and (hetero)­annulated systems while expanding the toolbox for dearomatization and oxidative cyclization.

## 4 Electrosynthesis of Six-Membered O-Heterocycles

Six-membered O-heterocycles are a particularly rich arena for electrosynthesis, where redox triggering enables not only ring construction but also rearrangements, ring expansions, and cascade-driven skeletal remodeling. Because these motifs are pervasive in drugs and bioactive natural products,^79,80^ electrochemical access is especially enabling. Across this section, cathodic pathways exploit electrogenerated nucleophiles and O_2_/superoxide chemistry, whereas anodic routes leverage mediated radicals and carbocationic intermediates.

### 4.1 Cathodic Processes

#### 4.1.1 Coumarins and Related δ-Lactones

Coumarins and related δ-lactones remain privileged scaffolds in medicinal chemistry, largely because their densely functionalized aromatic–lactone framework enables productive interactions with multiple biological targets and, consequently, a broad spectrum of pharmacological activities.^81^

Under cathodic conditions, controlled reduction of trichloroacetylated esters derived from 2-hydroxyacetophenones and trichloroacetyl chloride provides 3-chloro-4-substituted coumarins in good yields (Scheme 18).^82,83^ Mechanistically, the key step is an intramolecular addition of an electrogenerated carbanion onto the ketone functionality of the molecule. These transformations were typically conducted potentiostatically using dry MeCN/LiClO_4_ as SSE.

**Scheme 18:**
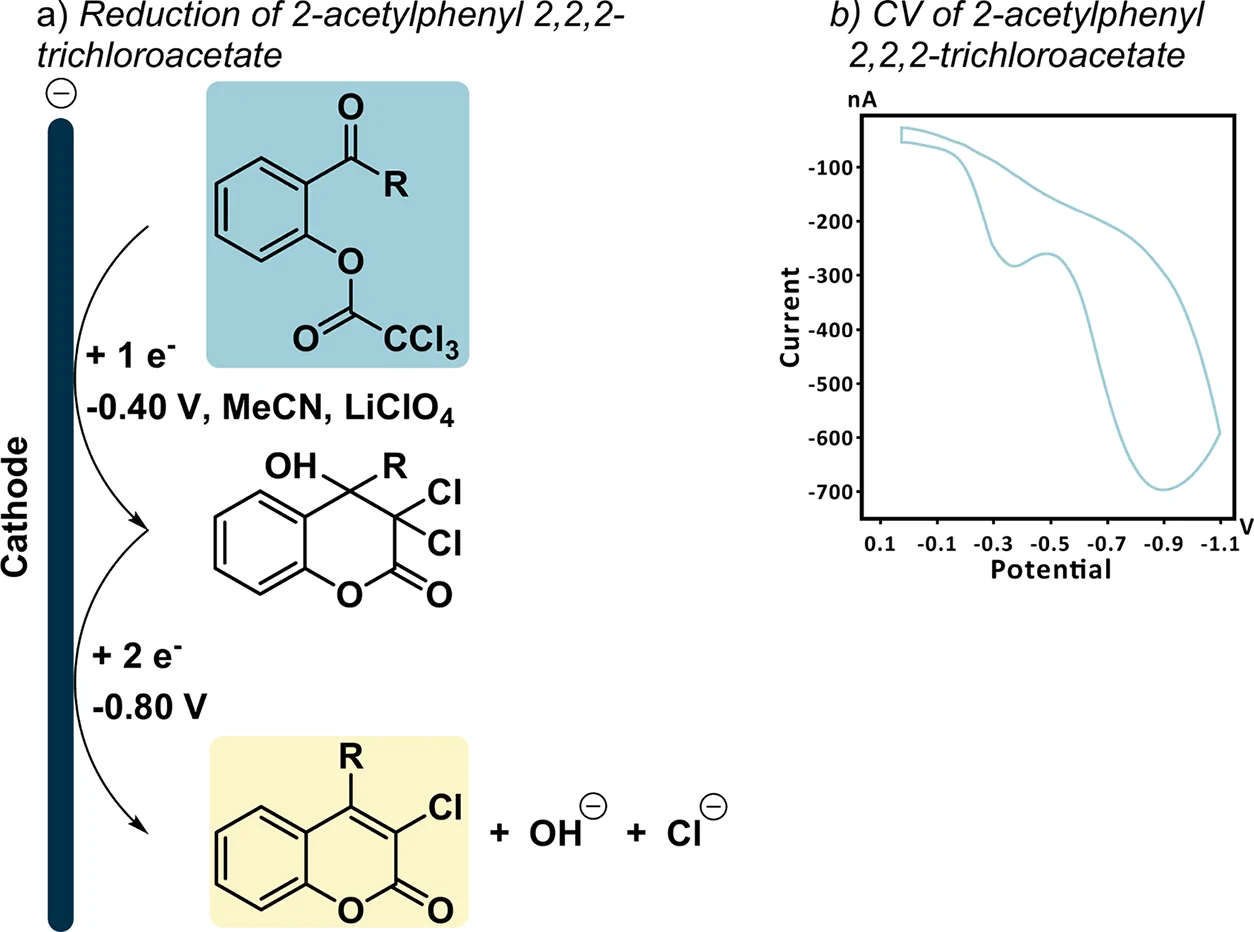
a) Cathodic Cyclization of Trichloroacetyl *o*-Hydroxyacetophenone Esters to 3-Chloro-4-Substituted Coumarins and b) CV of 2-Acetylphenyl 2,2,2-Trichloroacetate

Similarly, 3-chloro-benzocoumarins were obtained from trichlorinated esters of salicylaldehydes by reduction at – 1.0 V (vs SCE) in aprotic media.^84^ Related reductive procedure also enabled the one-step synthesis of α,α-dichloro-β-hydroxy-δ-lactones via electroreduction of aldol trichloroacetates, starting from readily available commercial building blocks.^85^

In a distinct mechanistic context, electrocatalytic addition of cyclic 1,3-ketoesters to isatins affords 4-hydroxy-3-substituted coumarins (chromen-2-ones) or, depending on substrate/conditions, pyran-2-ones with excellent efficiency (80–95% yield; 800–950% current efficiency).^86^

Oxygen can also act as a productive participant in cathodic lactone formation. Electroreductive conversion of 1,2-quinones to 6*H*-dibenzo­[*b,d*]­pyran-6-one and 2-benzopyran-1­(1*H*)-one was achieved in MeCN/LiClO_4_ (Scheme 19).^87^ In this scenario, homogeneous electron transfer from the quinone radical anion to molecular oxygen generates superoxide anion, which then engages in an electrochemical Baeyer–Villiger/Dakin-type sequence to furnish δ-lactones; carboxylic acids are often observed as side products. Collectively, these reactions position O_2_/superoxide as a broadly enabling electrochemical handle for δ-lactone formation, providing oxidative rearrangement chemistry without stoichiometric peroxides or chemical oxidants.

**Scheme 19:**
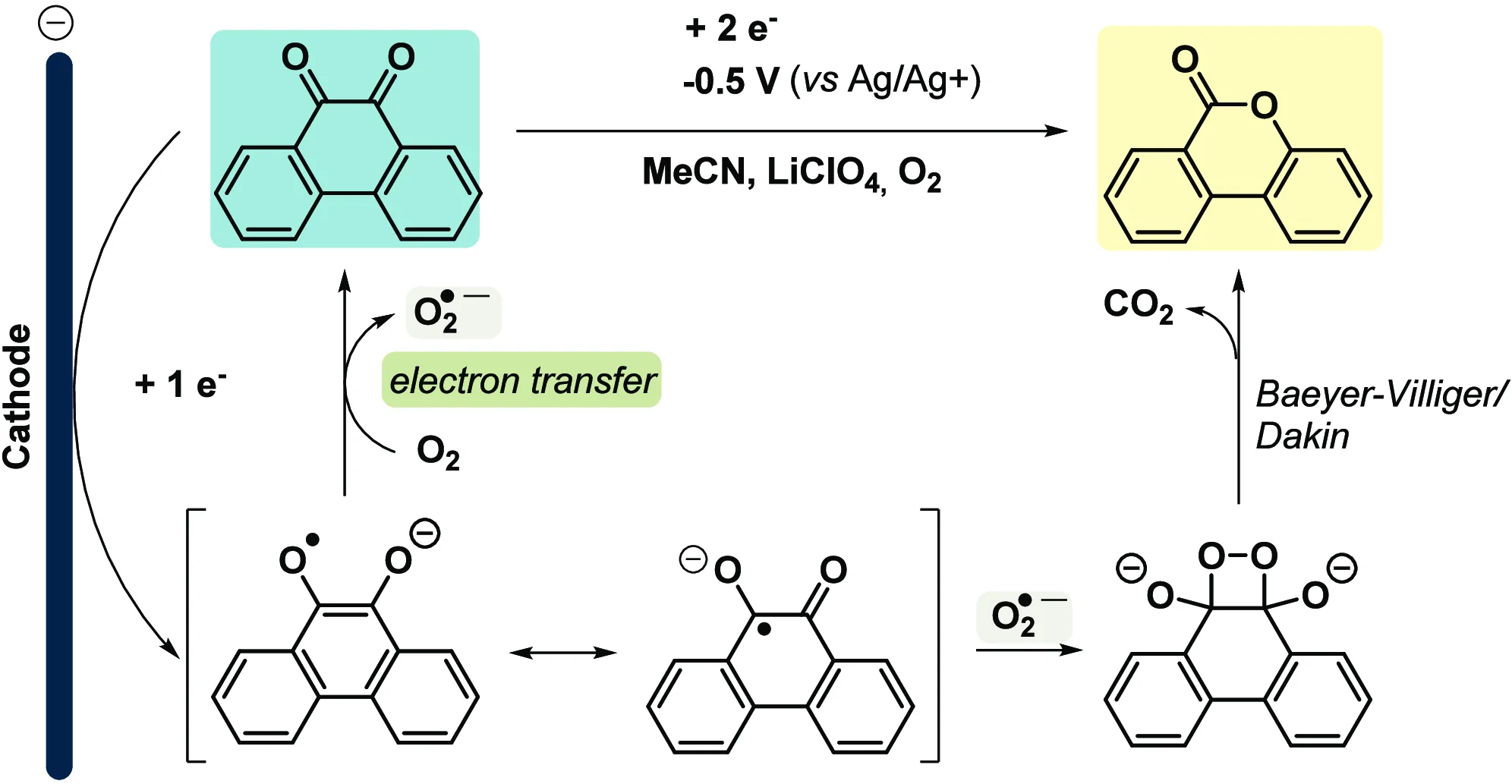
Superoxide-Mediated Electroreductive Baeyer–Villiger/Dakin Oxidation of 1,2-Quinones to δ-Lactones

A closely related “superoxide-enabled” ring expansion, a representative skeletal-editing event, was reported for 2,3-diphenyl-1*H*-inden-1-one: concomitant reduction of O_2_ and the indenone under potentiostatic control (−1.2 V vs Ag/Ag^+^) in MeCN/LiClO_4_ (SSE) at a graphite cathode delivers 3,4-diphenyl-1H-isochromen-1-one (44% yield).^88^ Product formation was rationalized by initial coupling of electrogenerated superoxide with the indenone radical anion (Scheme 20), followed by a Baeyer–Villiger-type rearrangement that effects ring expansion to the isocoumarin framework. This reaction exemplifies skeletal editing under electroreductive control, where access to radical anions and superoxide reprograms ring topology under mild conditions.

**Scheme 20:**
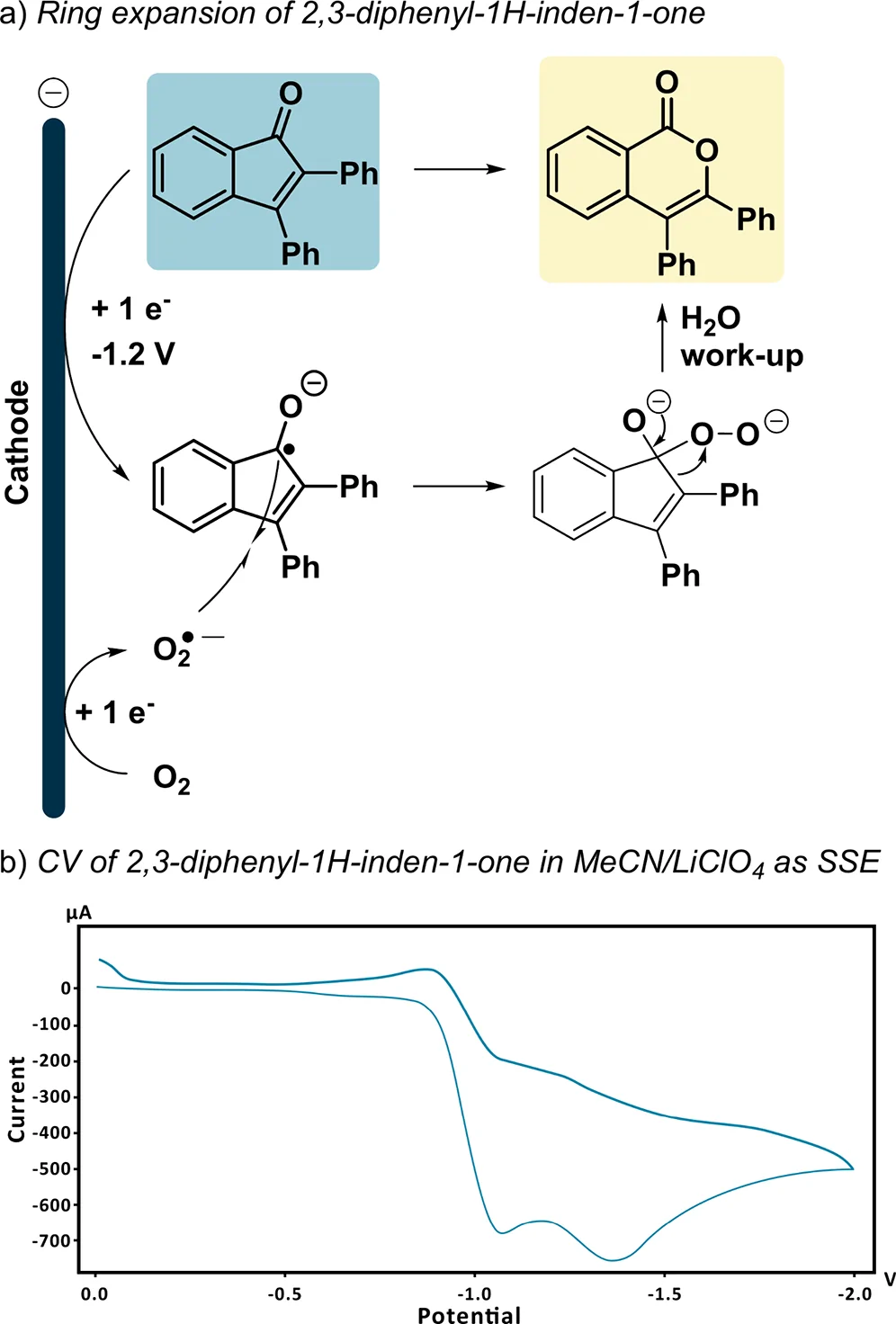
a) Superoxide-Enabled Electroreductive Ring Expansion of 2,3-Diphenyl-1*H*-inden-1-one to 3,4-Diphenyl-1*H*-isochromen-1-one and b) Cyclic Voltammetry of 2,3-Diphenyl-1*H*-inden-1-one in MeCN/LiClO_4_ as SSE

Recent NHC–CS_2_-mediated electrosynthesis of coumarin frameworks further underscores how tailored organic mediators can expand cathodic access to lactone-containing O-heterocycles beyond direct substrate reduction.^89^

In an EGB manifold, salicylaldehydes undergo a catalytic cascade with 4-hydroxy-6-methyl-2*H*-pyran-2-one to furnish 3-acetoacetylcoumarins in very good yields (Scheme 21).^3^ This process was performed in an undivided cell at an iron cathode using EtOH/NaBr as the SSE system, and exceptionally high current efficiencies (approaching ∼ 900%) were reported at current densities around 100 mA/cm^2^.

**Scheme 21:**
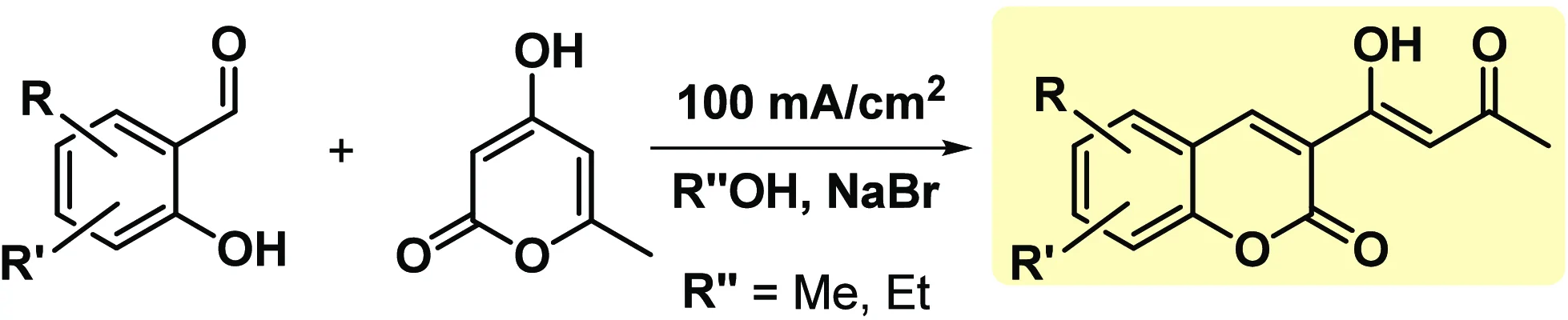
Electrogenerated-Base-Promoted Cascade Coupling of Salicylaldehydes with 4-Hydroxy-6-methyl-2*H*-pyran-2-one to 3-Acetoacetylcoumarins

Notably, these cathodic lactonizations illustrate how carbanion/EGB chemistry and O_2_/superoxide manifolds can deliver δ-lactones and ring expansions with high efficiency under potential control.

#### 4.1.2 1,3-Dioxanes and 1,3-Dioxines

Six-membered O,O-heterocycles such as 1,3-dioxanes and 1,3-dioxines are recurrent motifs in bioactive compounds and functional materials, and electrochemical methods provide complementary entries to these frameworks under mild, redox-controlled conditions. Their occurrence in bioactive natural products and functional materials has likewise motivated the development of efficient synthetic entries to these rings.^90^

Under cathodic conditions, 6,7-dihydrobenzo­[*d*]­[1,3]­dioxine-8-one was obtained from 1,2-cyclohexanedione by reductive electrolysis in CH_2_Cl_2_ using Et_4_NCl as SSE (Scheme 22).^91^ The transformation was rationalized to proceed through indirect reduction of dichloromethane, followed by radical coupling and an intramolecular nucleophilic substitution to close the six-membered heterocycle.

**Scheme 22:**
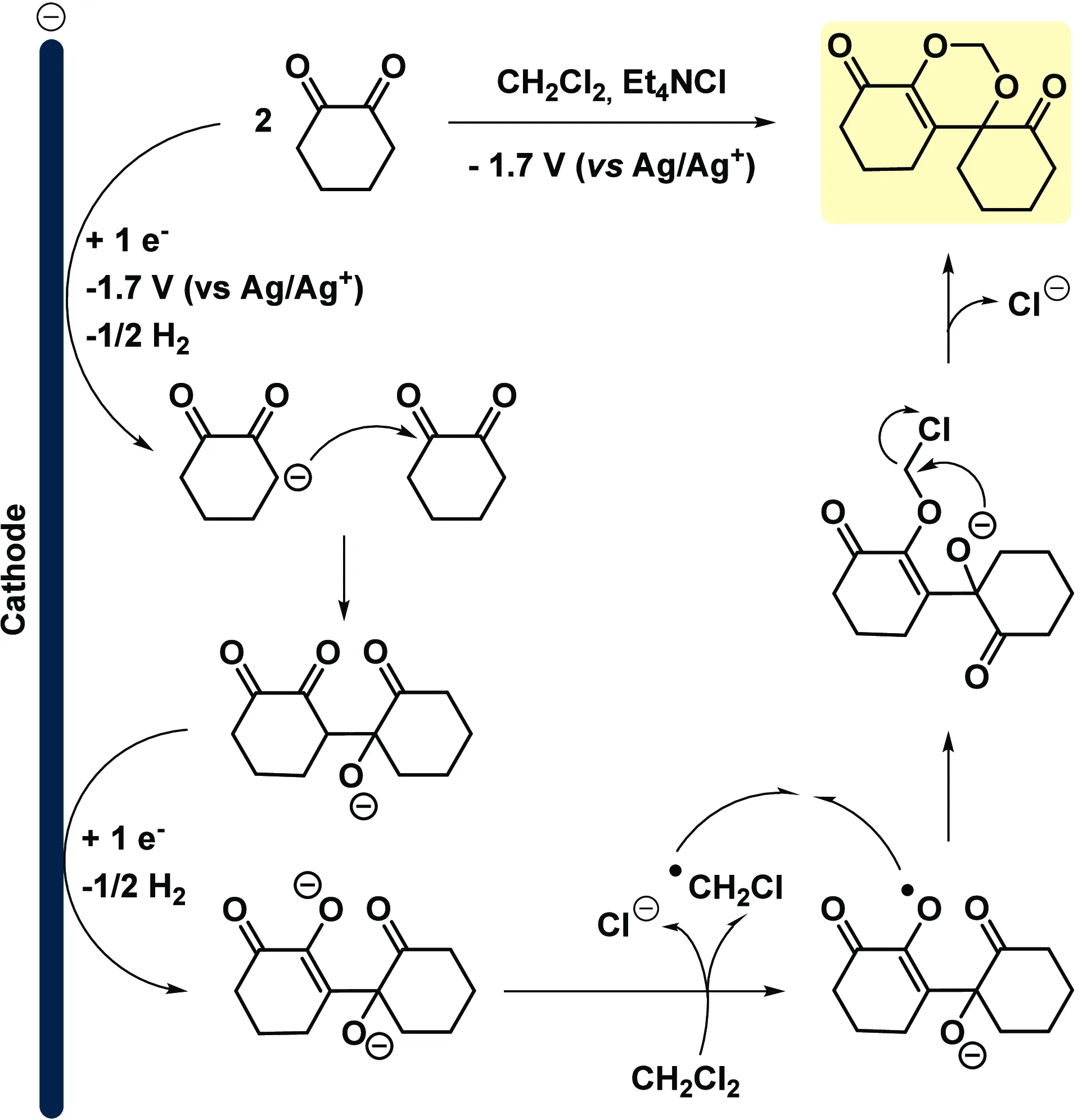
Cathodic Formation of 6,7-Dihydrobenzo­[*d*]­[1,3]­dioxine-8-one from 1,2-Cyclohexanedione via Indirect CH_2_Cl_2_ Reduction and Radical Coupling

Similarly, 2,3-dihydrobenzo­[1,2-*b*]­[1,4]­dioxins were formed upon electroreduction of ortho-quinones when 1,2-dichloroethane served as solvent.^39^ In this case, a 2 F/mol process generates a quinone-derived dianion that undergoes two successive nucleophilic substitutions on a fresh solvent molecule, ultimately delivering the dioxin framework.

These examples underscore a recurring electrosynthetic motif: indirect solvent activation (notably of chlorinated media) can supply reactive radical/electrophile equivalents that are captured by electrogenerated anions to forge O,O-heterocycles.

#### 4.1.3 Other Six-Membered O-Heterocycles Containing Nitrogen

Electroreduction of the C–Br bond in phenacyl bromide semicarbazones under highly dilute conditions in aprotic DMF/LiClO_4_ affords 3,7-diaryl-imidazo­[2,1-*b*]­[1,3,4]­oxadiazines in good yields (Scheme 23).^92^ The transformation was proposed to proceed via generation of a carbanion, followed by nucleophilic addition to the carbonyl group of a fresh substrate molecule, triggering cyclization to the six-membered O,N-heterocycle.

**Scheme 23:**
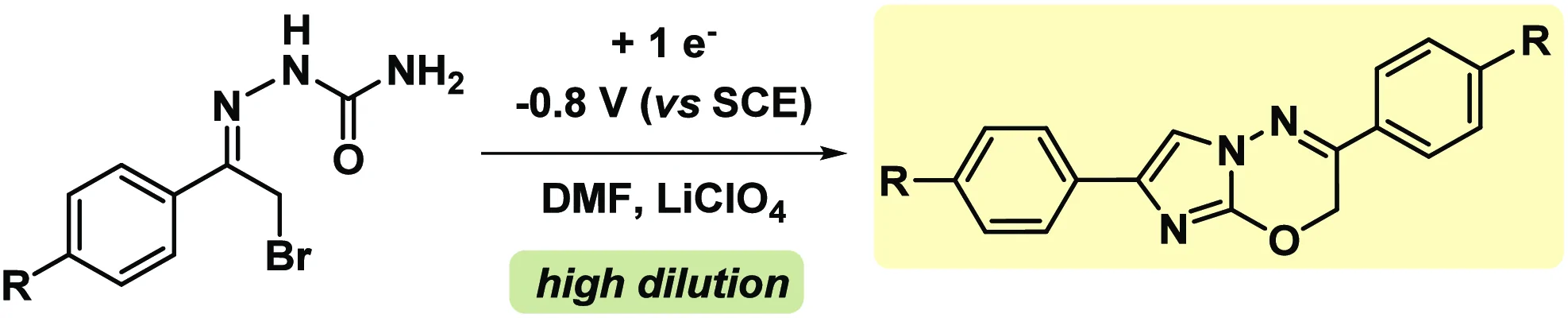
Electroreductive C–Br Cleavage of Phenacyl Bromide Semicarbazones Enabling 2*H*-Imidazo­[2,1-*b*]­[1,3,4]­oxadiazine Formation

Overall, cathodic C–X activation of suitably designed precursors offers a direct entry to six-membered O,N-heterocycles by coupling electrogenerated carbanions with intramolecular trapping and ring closure.

### 4.2 Anodic Processes

#### 4.2.1 Pyrans and Dioxines

Anodic methods have enabled efficient access to pyran- and dioxine-type frameworks through redox-controlled radical pathways. For instance, highly substituted dihydropyrano­[4,3-*b*]­indoles were prepared via an indirect anodic double oxidative [3 + 3] cycloaddition.^93^ This tandem radical-radical cross-coupling/6π-electrocyclization is mediated by readily available NaI, with O_2_ proposed to assist in base generation (or to act as a pre-base in the overall sequence). Anodic oxidation of enol ethers provides a direct entry to tetrahydropyran rings through radical-cation oxidative cyclization, further highlighting the value of anodic manifolds for pyran construction.^94^ Electrochemical oxidation of hispanolone furnished tetracyclic spiro-pyrans in >95% yield, showcasing anodic oxidation as a rapid complexity-building strategy.^95^

Similarly, organocatalyzed electrochemical coupling of alkenes with 1,2- and 1,3-diols provides 1,4-dioxanes and 1,4-dioxepanes, respectively, while hydrogen gas is generated at the cathode (Scheme 24).^96^ The use of a triarylamine electrocatalyst markedly improves the yields.

**Scheme 24:**
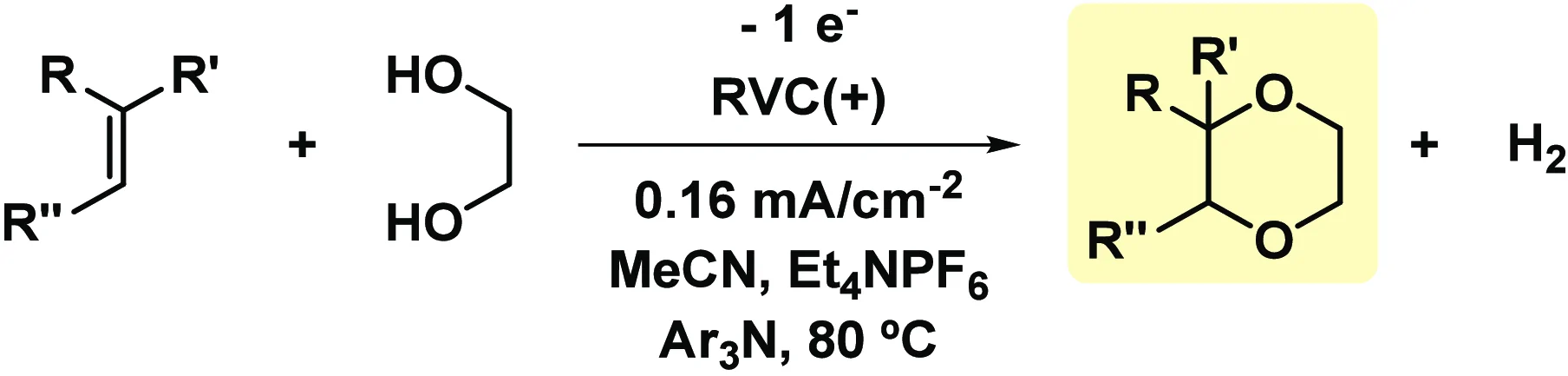
Organocatalyzed Anodic Dehydrogenative Annulation of Alkenes with Diols to 1,4-Dioxanes and 1,4-Dioxepanes

Together, these anodic annulations emphasize how mediated oxidation and dehydrogenative coupling can assemble pyran/dioxane frameworks efficiently, with electrocatalysts formats providing additional control.

#### 4.2.2 Terpenoid δ-Lactones

Terpenoid cycloalkanones were converted to δ-lactones by anodic oxidation at a Pt anode under neutral hydroalcoholic conditions using NaClO_4_ as SSE.^97^ The transformation is proposed to proceed via formation of a tertiary carbocation, followed by a 1,2-hydride shift and intramolecular cyclization to deliver the δ-lactone framework (Scheme 25).

**Scheme 25:**
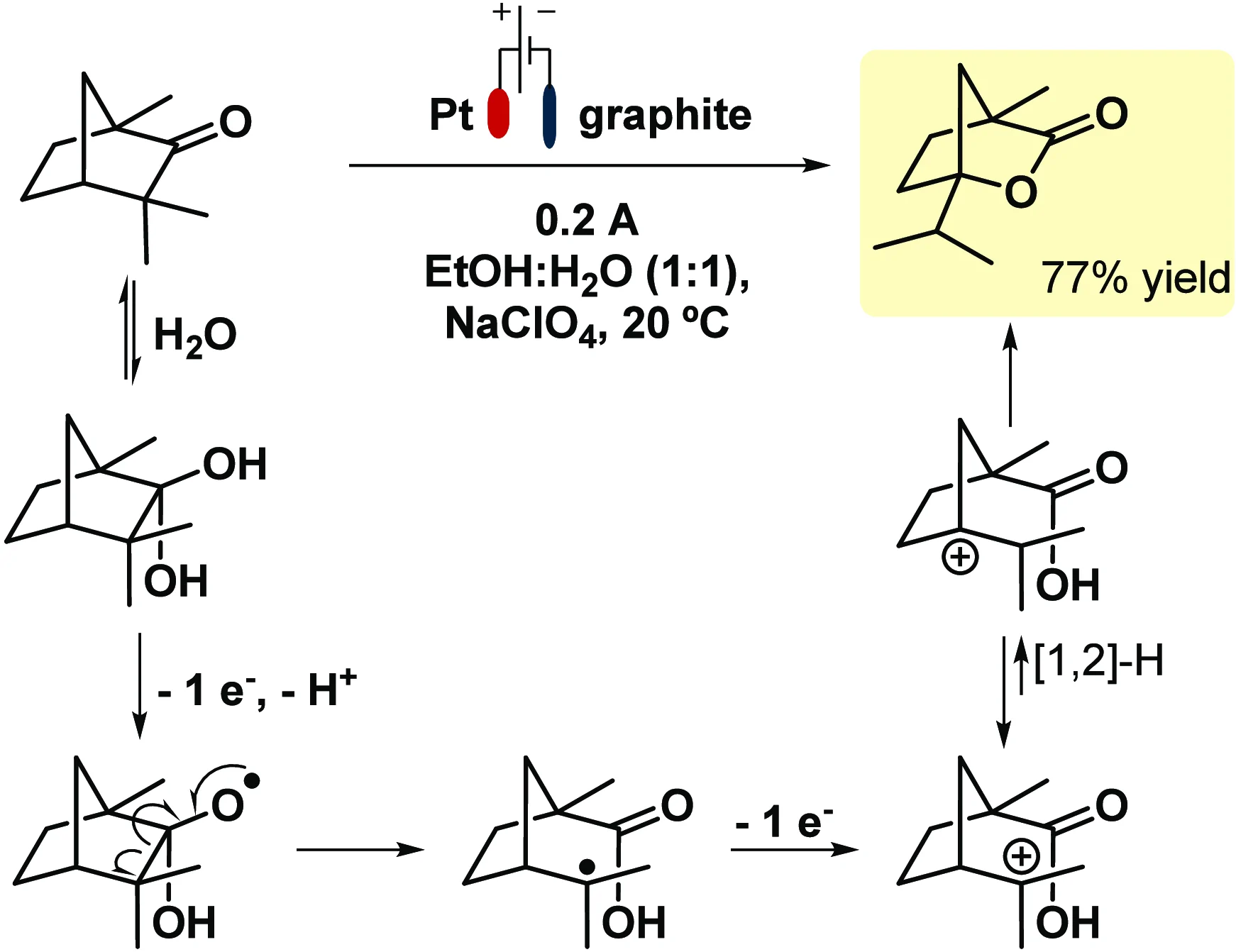
Anodic Oxidation of Terpenoid Cycloalkanones to δ-Lactones via a Carbocation/1,2-H Shift/Cyclization Sequence

A related anodic pathway was reported for 1-decalone, where oxidation triggers a carbocationic pathway culminating in ring contraction via a Wagner-Meerwein rearrangement (Scheme 26).^98^

**Scheme 26:**
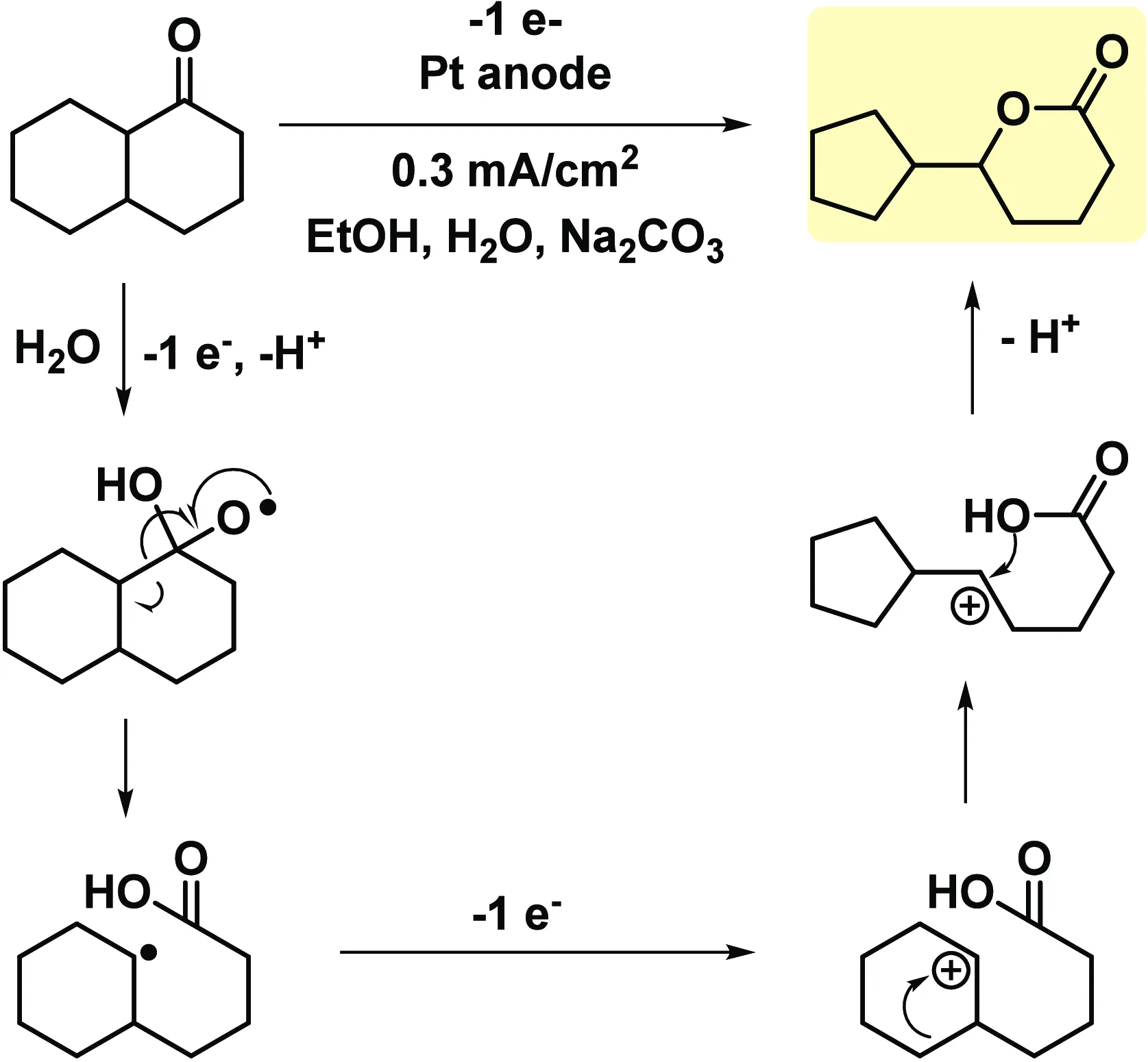
Anodic Oxidation of 1-Decalone: Carbocation-Mediated Ring Contraction via a Wagner-Meerwein Rearrangement

In contrast, six-membered lactones such as camphytolactone were also obtained from anodic oxidation of camphoric acid dicarboxylates in methanol at a Pt anode. In this case, product formation was attributed to an unexpected intramolecular radical coupling pathway (Scheme 27).^99^

**Scheme 27:**
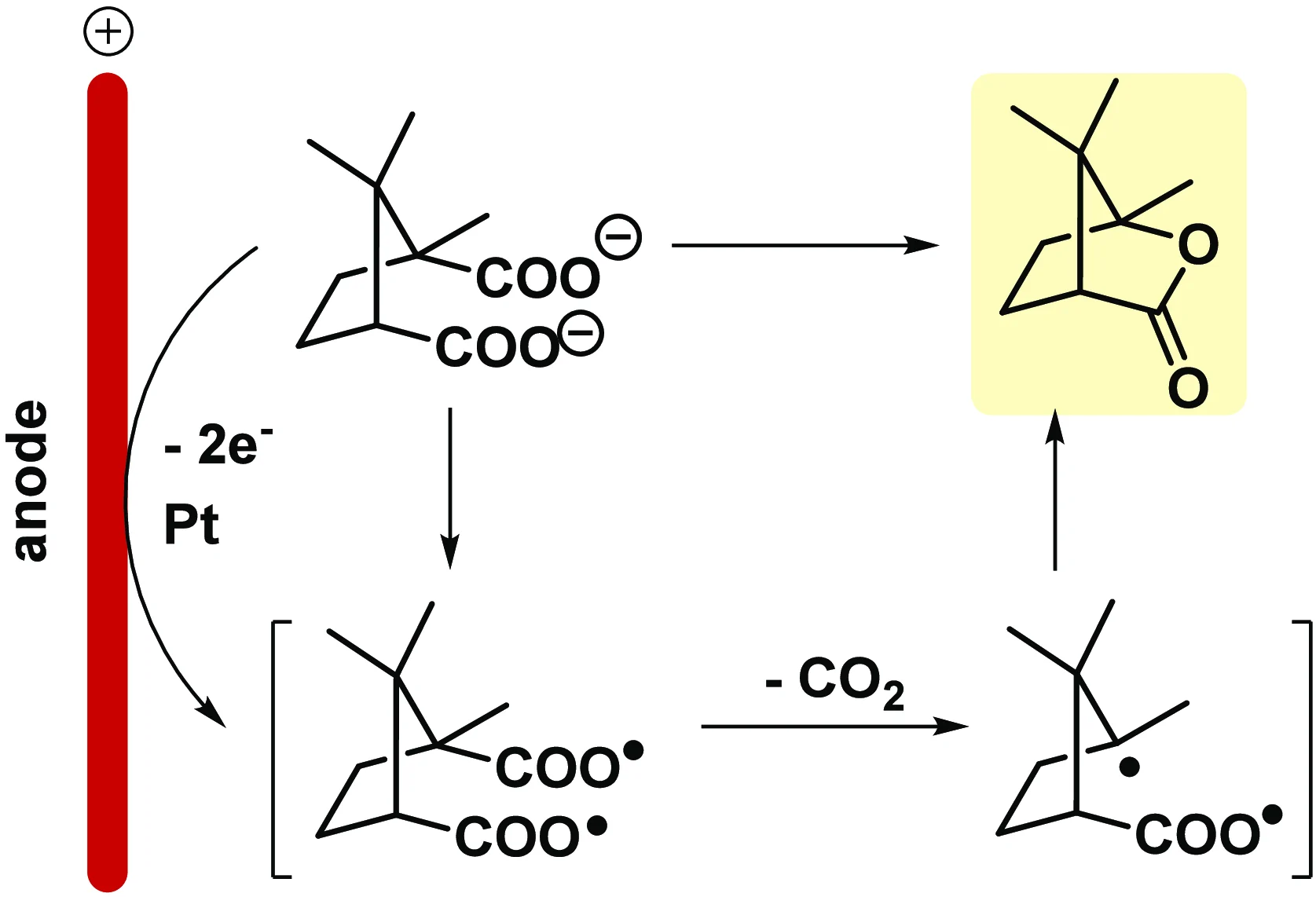
Anodic Oxidation of Camphoric Acid Dicarboxylates in MeOH: Radical Intramolecular Coupling En Route to Camphytolactone

These terpenoid examples highlight electrooxidation as a selective trigger for carbocationic rearrangements (shifts, contractions) and radical lactonization, enabling access to δ-lactones through pathways that are challenging to orchestrate with classical oxidants.

#### 4.2.3 Aromatic δ-Lactones

Indirect anodic oxidation of biphenyl-2-carboxylic acids in the presence of DDQ at a carbon anode provides 6*H*-benzo­[*c*]­chromen-6-ones in good yields.^100^ The intramolecular lactonization is proposed to proceed via an O-centered carboxyl radical that adds to the arene, followed by rearomatization to deliver the aromatic δ-lactone (Scheme 28).

**Scheme 28:**
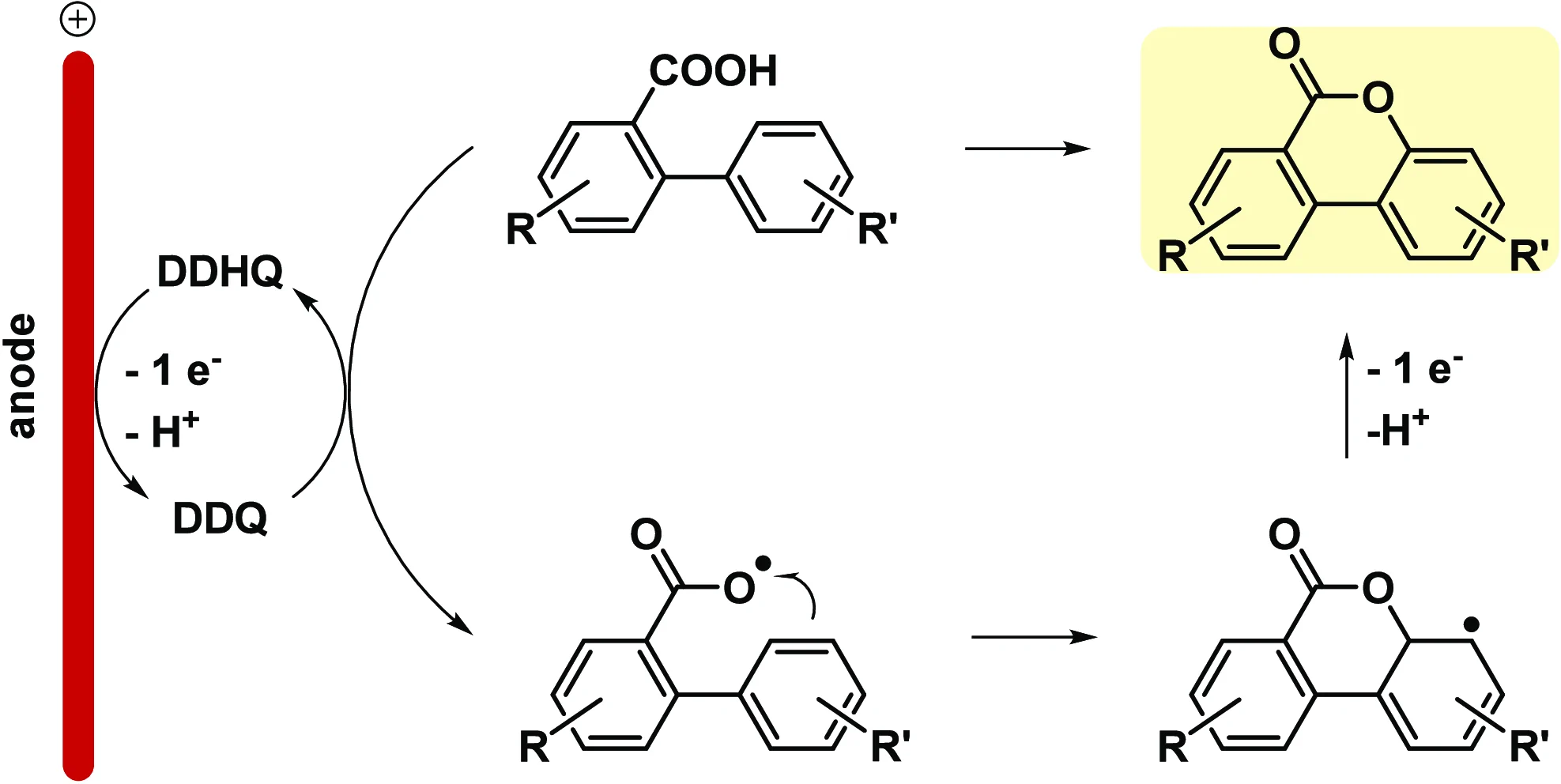
DDQ-Assisted Indirect Anodic Lactonization of Biphenyl-2-carboxylic Acids to 6*H*-Benzo­[*c*]­chromen-6-ones

This strategy was applied to the electrocatalyst-free synthesis of urolithin, a natural product of pharmacological interest, and was demonstrated on scale.^101^ The same general strategy was also extended to the formation of five-membered lactones, where a 1,5-HAT step is implicated in the product-forming sequence.^102^

A complementary entry to aromatic lactones relies on selenium-enabled anodic cyclizations. For example, anodic selenation/cyclization of a quinoidal lawsone derivative with diselenides at a Pt anode in MeCN/^*n*^Bu_4_NPF_6_ affords selanyl-2-phenyl-3,4-dihydro-2*H*-benzo­[*h*]­chromene-5,6-diones in moderate-to-good yields a seleniranium cation intermediate was proposed (Scheme 29a).^103^ Similarly, electrooxidation of o-(1-alkynyl)­benzoates with diaryl diselenides under continuous-flow (microreactor) conditions furnishes isocoumarin derivatives, with electrogenerated arylselenium radicals suggested to promote oxidative intramolecular cyclization (Scheme 29b).^104^ This approach offers a metal-free, oxidant-free platform for accessing biologically relevant isocoumarins. The microreactor format underscores the natural fit between electrosynthesis and flow chemistry, where residence-time control and enhanced mass transfer can preserve selectivity in radical/cationic cyclizations.

**Scheme 29:**
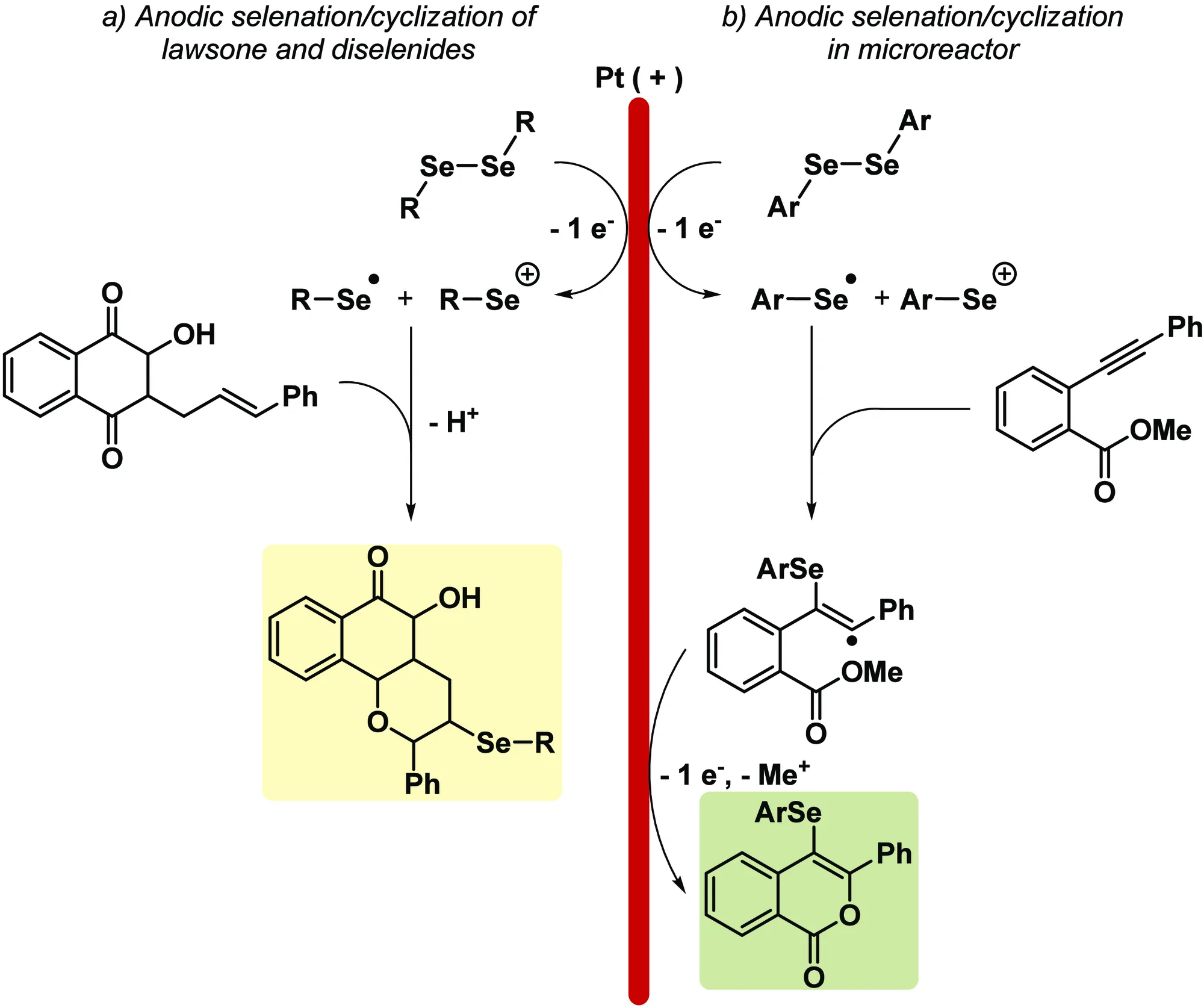
a) Anodic Selenation/Cyclization of a Lawsone-Derived Quinone with Diselenides via a Seleniranium Intermediate and b) Microreactor Anodic Oxidative Cyclization of o-(1-Alkynyl)­benzoates with Diaryl Diselenides to Isocoumarins

Isocoumarins were also prepared via anodic oxidation of alkenes, enabling intramolecular C–H acyloxylation through nucleophilic cyclization assisted by trifluoroacetate generated at the cathode.^105^

Overall, indirect anodic lactonization and selenium-enabled oxidative cyclizations demonstrate how anodic radical and cationic intermediates can be harnessed to construct aromatic δ-lactones and isocoumarins under metal-free, oxidant-free conditions.

#### 4.2.4 Six-Membered O-Heterocycles Containing Nitrogen

Intramolecular anodic aromatic C–H oxygenation of N-benzylamides enables access to 4*H*-1,3-benzoxazines in moderate-to-good yields at room temperature using MeCN/^*n*^Bu_4_NClO_4_ as SSE (Scheme 30).^106^

**Scheme 30:**
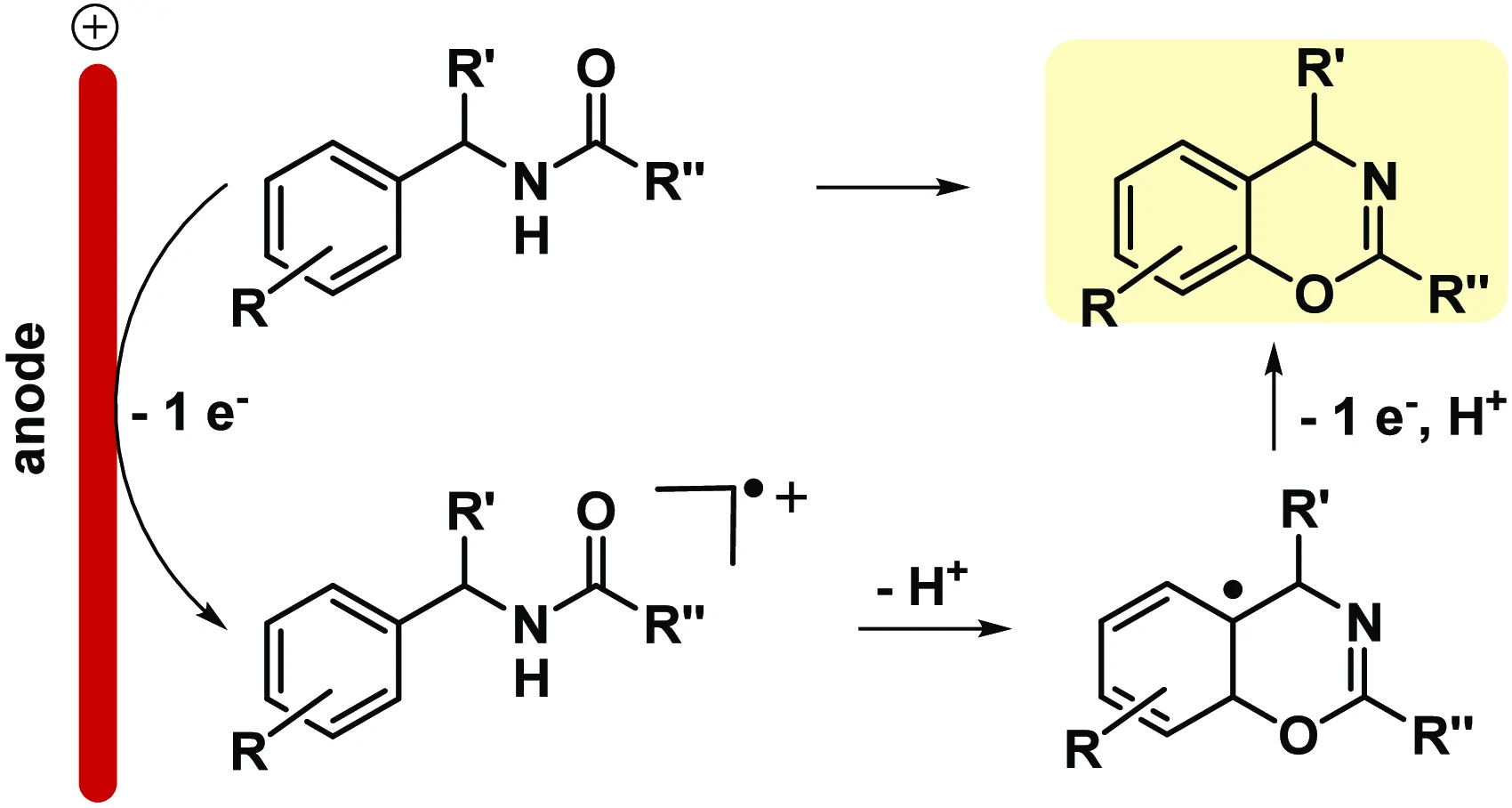
Intramolecular Anodic Aromatic C–H Oxygenation of *N*-Benzylamides to 4*H*-1,3-Benzoxazines

Benzo­[*c*]­[1,2]­oxazine scaffolds were also synthesized via anode-selective electrochemical oxidation of hydroxamic acids in the presence of olefins.^107^ This method exhibits high regio- and stereoselectivity and shows broad functional-group tolerance, including compatibility with complex feedstocks such as terpenes, peptides, and steroids. In addition, sulfonated 4*H*-3,1-benzoxazines can be formed through a metal-free electrochemical radical cascade cyclization of styrenyl amides with sulfonylhydrazines, forging the key C–O bond with broad substrate scope (Scheme 31).^108^

**Scheme 31:**
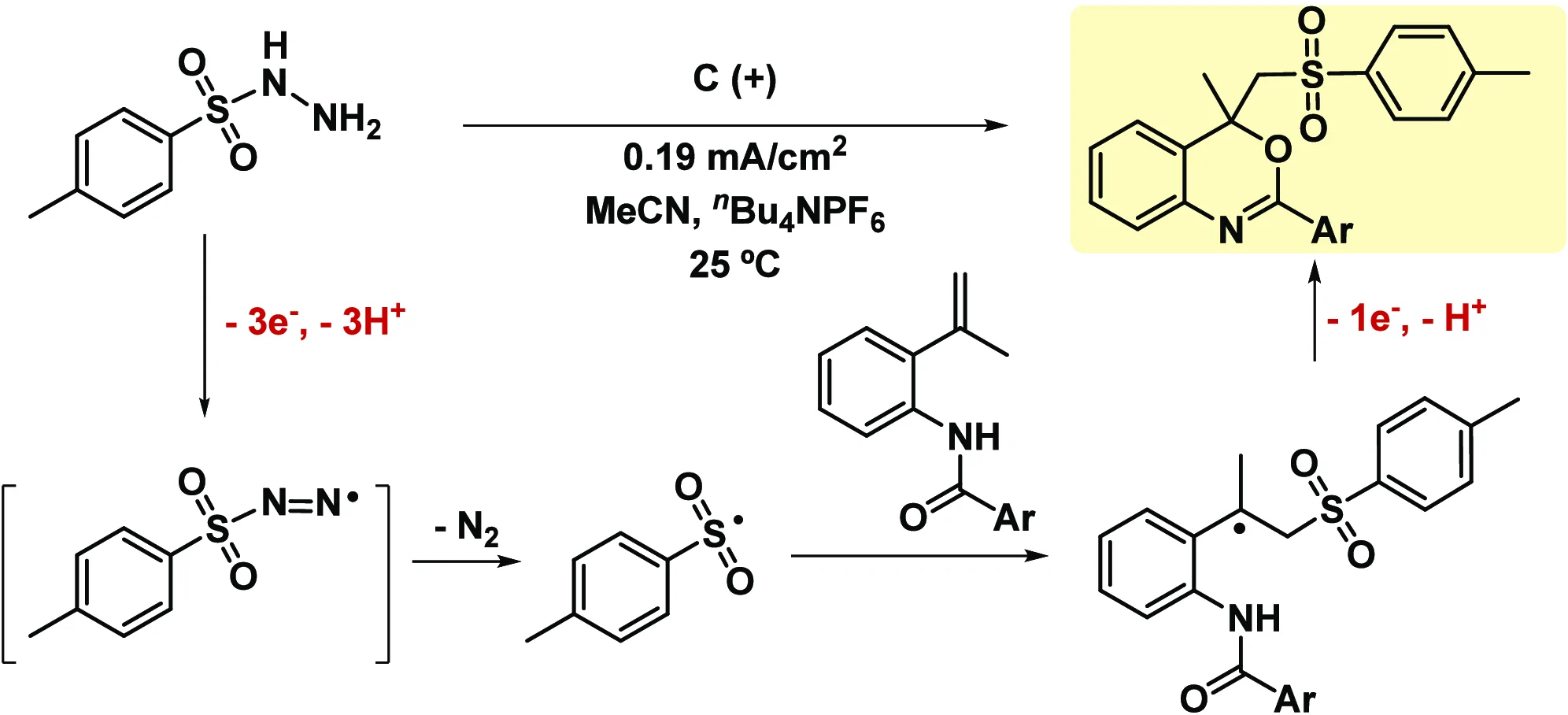
Metal-Free Electrochemical Radical Cascade Cyclization of Styrenyl Amides with Sulfonylhydrazines to Sulfonated 4*H*-3,1-Benzoxazines

Benzoxazinone frameworks were accessed through a 2-fold electrochemical C–H amination of activated arenes under metal-free conditions.^109^ The protocol relies on a two-step electrolysis (3.4 F at 5 mA/cm^2^, followed by 2.1 F at 10 mA/cm^2^) and is applicable to medicinally relevant targets; mechanistic proposals invoke a dicationic Zincke-type intermediate (Scheme 32).

**Scheme 32:**
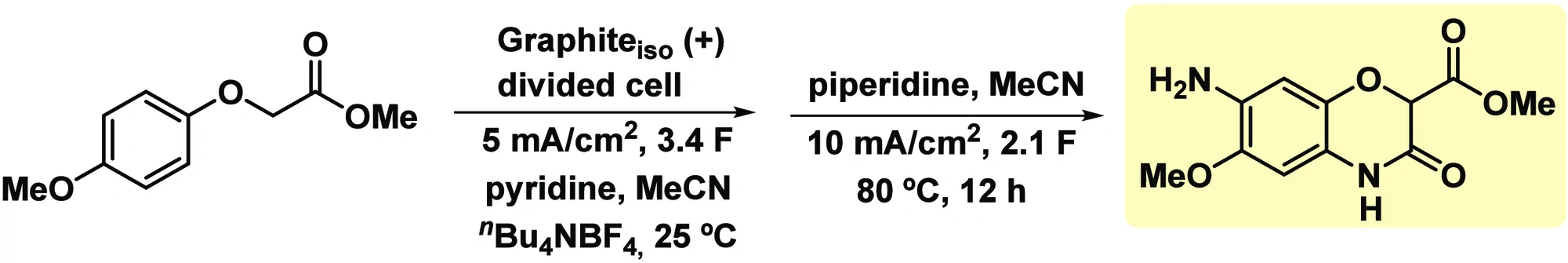
Two-Step Electrochemical C–H Amination to Benzoxazinones via a Dicationic Zincke-Type Intermediate

Beyond O,N systems, 1,4-benzoxathiins are efficiently assembled in a modular flow electrolysis cell via oxidative intramolecular dehydrogenative C–S coupling (Scheme 33).^110^ Finally, sultone derivatives can be obtained by anodic C–H functionalization of 2-arylbenzene sulfonates in MeCN/H_2_O with LiClO_4_ at a Pt anode (undivided cell, heating, 10 mA).^111^

**Scheme 33:**
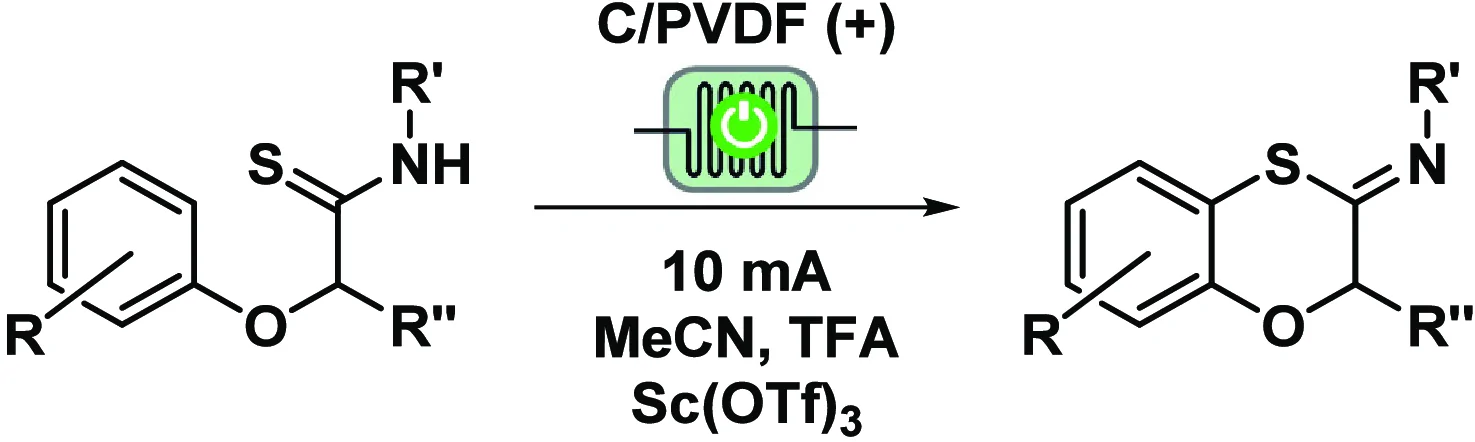
Flow-Electrochemical Oxidative Intramolecular Dehydrogenative C–S Coupling to 1,4-Benzoxathiins

Collectively, these methods show how anodic C–H functionalization and oxidative dehydrogenative coupling provide streamlined access to O,N and O,S six-membered heterocycles, with paired/flow electrolysis offering clear opportunities for late-stage diversification.

## 5 Conclusions and Future Outlook

Organic electrosynthesis has emerged as a powerful strategy for constructing oxygen heterocycles under mild and sustainable conditions, often replacing stoichiometric oxidants or reductants with electrons. Across the examples discussed here, electrochemistry enables not only ring formation but also ring expansion, rearrangement, and skeletal editing. Just as importantly, the chemistry surveyed reveals a field with substantial room for creative growth: many of the most successful transformations already show how finely tuned redox control, solvent/supporting electrolyte effects, and electrode choice can be translated into precise control over reactivity and selectivity. As these relationships become better understood, the prospect of extending such logic across broader O-heterocyclic families becomes especially compelling. In this sense, electrosynthesis stands not simply as a greener alternative to classical methods, but as an increasingly inspiring platform for discovering new reactivity and reimagining how oxygen heterocycles can be built, edited, and deployed.
